# ZR^2^ViM: a recursive vision Mamba model for boundary-preserving medical image segmentation

**DOI:** 10.3389/fbinf.2026.1768786

**Published:** 2026-03-04

**Authors:** Caijian Hua, Caorong Xiang, Liuying Li, Xia Zhou

**Affiliations:** 1 School of Computer Science and Engineering, Sichuan University of Science and Engineering, Yibin, China; 2 Traditional Chinese Medicine Department, Zigong First People’s Hospital, Zigong, China

**Keywords:** boundary preservation, deep learning, medical image segmentation, state space models, vision mamba, zigzag scanning

## Abstract

**Introduction:**

Medical image segmentation is fundamental to quantitative disease analysis and therapeutic decision-making. However, constrained by limited computational resources, existing deep learning methods often struggle to simultaneously model long-range dependencies and preserve boundary precision, particularly when delineating structures with complex morphology or blurred edges.

**Method:**

To overcome these challenges, we propose ${\text{ZR}}^{2}$ ViM, a recursion-enhanced visual state space model designed for medical image segmentation. ${\text{ZR}}^{2}$ ViM augments the Vision Mamba framework with a Zigzag Recursive Reinforced ( ${\text{ZR}}^{2}$ ) Block that incorporates Stacked State Redistribution (SSR) and a Nested Recursive Connection (NRC). The NRC employs dual inner and outer pathways to iteratively fuse local details with global context while preserving 2D spatial adjacency. Furthermore, a Cross-directional Zigzag WKV (CZ-WKV) module executes multi-step recursive updates along multiple zigzag trajectories, injecting spatial directional information via Quad-Directional Token Shift (Q-Shift) directional priors. Collectively, these mechanisms mitigate serialization-induced banding artifacts and enhance the representation of fine, elongated, and low-contrast structures, all while maintaining near-linear computational complexity.

**Results:**

Comprehensive evaluations across four medical imaging domains—spanning dermatoscopic images, breast ultrasound, colorectal polyps, and abdominal multi-organ CT—on five public datasets demonstrate that ${\text{ZR}}^{2}$ ViM consistently outperforms representative convolutional, attention-based, and visual state space architectures in region consistency and boundary localization. Notably, ${\text{ZR}}^{2}$ ViM achieves a 2.15 mm reduction in the HD95 on the Synapse multi-organ CT dataset relative to the CC-ViM baseline, substantiating its superior capability for precise, clinically relevant boundary delineation.

**Conclusion:**

The ${\text{ZR}}^{2}$ ViM framework delivers accurate, boundary-preserving segmentation across diverse imaging modalities and anatomically complex structures, achieving these gains with near-linear computational complexity. These findings demonstrate that ${\text{ZR}}^{2}$ ViM offers a robust and efficient solution for medical image analysis, establishing a promising foundation for advanced clinical and research applications.

## 1 Introduction

Medical image segmentation is critical for disease screening, lesion localization, preoperative planning, and treatment efficacy assessment, as segmentation accuracy directly influences subsequent quantitative analysis and clinical decision-making (Litjens et al., 2017; Chan et al., 2020). Unlike natural images, medical images are often characterized by high resolution, low contrast, and irregular boundaries. These challenges are compounded by high annotation costs and limited sample sizes, complicating three core tasks: modeling long-range dependencies, preserving boundary continuity, and ensuring robust inference, particularly for low-contrast images and elongated structures (Isensee et al., 2021; Wang et al., 2021). Consequently, developing a unified framework that achieves both high precision and computational efficiency remains a central challenge in medical image segmentation.

Traditional convolutional neural networks (CNNs) rely on local convolution kernels and fixed receptive fields, a design that limits their ability to encode global anatomical context. Although architectures like UNet and its variants (Ronneberger et al., 2015; Zhou et al., 2018; Oktay et al., 2018; Xiao et al., 2018) enhance multi-scale representations through encoder–decoder designs and skip connections, CNNs have inherent limitations in modeling long-range dependencies. They therefore often produce fragmented predictions or blurred boundaries, especially when segmenting large, weakly contrasted, or elongated anatomical structures. In contrast, Transformer-based models leverage global self-attention to model long-range dependencies more effectively. Architectures such as TransUNet (Chen et al., 2024) and Swin-Unet (Liu et al., 2021), inspired by the Vision Transformer (ViT) (Han et al., 2023), have demonstrated strong performance in medical imaging. However, the quadratic computational complexity of self-attention (Han et al., 2023) results in prohibitive memory costs and inference latency as image resolution increases. Furthermore, their lack of convolutional inductive bias can compromise robustness on small medical datasets and degrade the localization of fine-grained boundaries. Thus, despite their global modeling strengths, Transformers face intrinsic scalability and stability limitations in this domain.

Recently, Mamba-based state space models (SSMs) (Gu and Dao, 2024) have emerged as a compelling alternative, offering near-linear complexity and efficient long-sequence modeling. The Vision Mamba (ViM) (Liu Y. et al., 2024) extends SSMs to the visual domain by incorporating multi-directional selective scanning. Nevertheless, these models still flatten 2D feature maps into 1D sequences, a process that disrupts the intrinsic 2D spatial adjacency and directional continuity. This serialization weakens the model’s ability to represent complex boundaries and slender structures, limiting segmentation performance on challenging targets. Another approach, the Receptance Weighted Key–Value (RWKV) model (Zhou et al., 2023; Duan et al., 2024), integrates recurrent updates with key-value interactions and has emerged as a potential successor to Transformers due to its linear computational complexity and robust long-range dependency modeling. However, its direct application to segmentation reveals critical limitations: while it excels at modeling long-range dependencies, it lacks mechanisms for explicit modeling of local fine-grained features and 2D geometric adjacency. These shortcomings underscore the necessity of integrating explicit 2D structural priors into recurrent SSMs for robust medical image segmentation.

This analysis raises a central question: how can two-dimensional spatial adjacency and directional continuity be restored within a vision state space model, while maintaining near-linear complexity and enabling effective interaction between local details and global context? To address this, we introduce ${\text{ZR}}^{2}$ ViM (Zigzag Recursive Reinforced Vision Mamba), a novel architecture that restructures the foundational ViM framework. The fundamental component of our model is the Zigzag Recursive Reinforced ( ${\text{ZR}}^{2}$ ) Block, which implements a Selective State Recurrence (SSR) operator within a Nested Recurrent Cell (NRC). The NRC features two parallel paths, NRC-Inner and NRC-Outer, to process features at different scales. Within this block, we propose the Cyclic Zigzag Weighted Key Value (CZ-WKV) attention mechanism, which employs an expandable, multi-directional cyclic zigzag scan to aggregate contextual information. By integrating CZ-WKV into the nested recurrent architecture, SSR achieves superior spatial alignment and directional robustness compared to the unidirectional scanning in conventional SSMs. A residual connection from the NRC-Inner to the NRC-Outer path ensures the reliable injection of fine-grained details, enhancing boundary delineation while preserving the model’s near-linear computational complexity.

The main contributions of this work are:

• A novel segmentation architecture, ${\text{ZR}}^{2}$ ViM, that enhances visual state space models with mechanisms for spatial continuity and global context while maintaining near-linear complexity.
• The core SSR operator, implemented via a NRC, which jointly models intra-patch details and inter-patch context. Its extensible zigzag scan explicitly restores 2D spatial and directional priors.
• The CZ-WKV attention mechanism, which efficiently aggregates multi-directional context in linear time within the recursive scanning framework, balancing global dependency modeling with spatial continuity.
• Comprehensive validation across multiple public datasets demonstrating that ${\text{ZR}}^{2}$ ViM significantly outperforms state-of-the-art methods—particularly for images with complex boundaries, low contrast, and slender structures—at a low computational cost.

## 2 Related work

### 2.1 Medical image segmentation

#### 2.1.1 CNN-based methods

CNNs have long been the cornerstone of medical image segmentation, prized for their powerful local feature extraction capabilities. The seminal UNet (Ronneberger et al., 2015) architecture introduced a symmetric encoder–decoder design with skip connections, a structure that proved highly effective for fusing shallow, fine-grained details with deep semantic features, especially on limited medical datasets. However, the original UNet design offered limited interaction between features at different semantic scales. Subsequent variants sought to address these shortcomings. For instance, UNet++(Zhou et al., 2018) introduced dense skip connections to bridge this “semantic gap” and improve feature fusion, yet it often failed to precisely delineate targets with complex boundaries. Similarly, Attention-UNet (Att-UNet) (Oktay et al., 2018) incorporated attention gates to suppress background noise and focus on salient regions, thereby improving accuracy for intricate structures. To enable the training of deeper, more powerful models, other frameworks integrated residual connections, inspired by ResNet (He et al., 2016), to mitigate the vanishing gradient problem and enhance feature representation in deep layers. Despite these advances, all CNN-based architectures are fundamentally constrained by the local nature of the convolution operation, which limits their ability to model long-range spatial dependencies. This intrinsic locality often results in inconsistent segmentation of large organs or blurred predictions along complex boundaries. While techniques like dilated convolutions expand the receptive field, they typically do so at the cost of sacrificing fine-grained local detail (Han et al., 2023).

#### 2.1.2 Transformer-based methods

To overcome the intrinsic locality of CNNs, Transformers leverage a global self-attention mechanism to model long-range spatial dependencies (Shamshad et al., 2023). The ViT (Han et al., 2023) pioneered the use of pure Transformer architectures for vision tasks, but its direct application to medical imaging is challenging due to the data-hungry nature of self-attention and the typically small scale of medical datasets. Subsequent innovations like the Swin Transformer (Liu et al., 2021) addressed the prohibitive computational cost of global attention by introducing a hierarchical, windowed self-attention mechanism that scales linearly with image size. This efficient design was later adapted into U-shaped architectures such as Swin-UNet (Cao et al., 2022), creating a pure Transformer-based model for segmentation. A parallel research direction sought to combine the strengths of both paradigms in hybrid CNN-Transformer models. These architectures aim to retain the robust local feature extraction of CNNs while incorporating the global context modeling of Transformers. Prominent examples include TransUNet (Chen et al., 2024), which embeds a Transformer in the encoder of a U-Net to capture global context; UNETR (Hatamizadeh et al., 2022), which pairs a Transformer encoder with a convolutional decoder for 3D volumetric segmentation; and TransFuse (Zhang et al., 2021), which uses a dual-branch structure to fuse features from parallel CNN and Transformer backbones. Despite these architectural innovations, a fundamental bottleneck remains: the quadratic computational complexity of standard self-attention. This leads to prohibitive memory usage and inference latency on the high-resolution images common in clinical practice. Furthermore, the reduced inductive bias of Transformers compared to CNNs often necessitates extensive pre-training and can compromise model stability and performance on small datasets, particularly in localizing complex boundaries (Han et al., 2023; Liu et al., 2021).

#### 2.1.3 Mamba-based methods

SSMs have recently emerged as a powerful alternative to Transformers, offering comparable long-range dependency modeling at near-linear computational complexity. The foundational Mamba model (Gu and Dao, 2024) achieves this efficiency through a selective state space mechanism (S6), but its unidirectional processing limits its native awareness of 2D spatial structures. To adapt this paradigm for vision, the ViM (Liu Y. et al., 2024) introduced a multi-directional scanning process (SS2D) that transforms image features into complementary 1D sequences, establishing a blueprint for visual SSMs. This foundational work prompted a rapid proliferation of Mamba-based architectures for medical segmentation. Many of these, such as U-Mamba (Bao et al., 2025) and VM-UNet (Ruan et al., 2024), integrated Mamba blocks into established U-shaped frameworks to enhance their feature encoders. Other efforts have focused on improving computational efficiency for resource-constrained environments (e.g., LightM-UNet (Wu et al., 2024)) or leveraging pre-training to improve generalization (e.g., Swin-U-Mamba (Liu J. et al., 2024)). However, a critical limitation pervades these first-generation visual SSMs. They all rely on flattening 2D feature maps into 1D sequences for processing. This serialization fundamentally disrupts the intrinsic 2D spatial adjacency and directional relationships inherent in images. As a result, their ability to model complex boundaries and preserve the continuity of slender anatomical structures is compromised, hindering their performance on challenging segmentation targets (Liu Y. et al., 2024). This architectural flaw underscores the need for a new approach that can process visual information in its native 2D context.

### 2.2 Visual state space modeling and recursive enhancement

Visual state space models (Gu and Dao, 2024), exemplified by Mamba and ViM (Liu Y. et al., 2024), have shown considerable promise for medical image segmentation but also face inherent limitations. To address these, recent architectures have incorporated recursive mechanisms to enhance long-range information propagation and directional awareness. A prominent example is the RWKV model (Zhou et al., 2023), which integrates linear-time recursive updates with key–value interactions. This design achieves low computational cost and stable long-range dependency modeling, and its efficacy has been proven in general-purpose vision and natural language tasks, such as Vision-RWKV (Duan et al., 2024). However, a critical challenge arises when applying RWKV directly to medical image segmentation. Its strength in modeling global context often comes at the expense of capturing fine-grained local spatial continuity. This limitation hinders its ability to accurately delineate complex anatomical structures, a fundamental requirement for clinical applications (Zhou et al., 2023; Duan et al., 2024).

To address this limitation, we introduce ${\text{ZR}}^{2}$ ViM, a model that advances the ViM-based (Liu Y. et al., 2024) U-shaped architecture through two synergistic innovations: a nested recursive block and a multi-directional zigzag space-mixing mechanism. This design explicitly restores 2D spatial adjacency and strengthens directional context aggregation, enabling the simultaneous modeling of fine-grained local details and global context with near-linear complexity. Consequently, ${\text{ZR}}^{2}$ ViM excels at accurately delineating complex boundaries, slender structures, and multi-scale anatomical regions within medical images. Critically, while maintaining a compact parameter footprint and near-linear computational cost, ${\text{ZR}}^{2}$ ViM outperforms leading convolutional, Transformer-based, and state space models across diverse public medical image segmentation benchmarks.

## 3 Methods

### 3.1 Preliminary knowledge

SSMs describe how an input sequence drives the evolution of a hidden state and generates an output sequence. In the continuous-time case, a first-order linear SSM can be written as in Equation 1:

$$\begin{array}{cc} h^{\prime }\left(t\right)=\; & Ah\left(t\right)+Bx\left(t\right)\\ y\left(t\right)=\; & Ch\left(t\right) \end{array}$$ (1)

where $x\in R^{L}$ is the input sequence, $h\left(t\right)\in R^{N}$ is the latent state, y(t) is the output, and $A\in R^{N\times N},B\in R^{N\times 1},C\in R^{1\times N}$ are learnable parameter matrices.

For use in deep-learning models, this continuous-time system is usually converted into a discrete-time form. Using Zero-Order Hold (ZOH) with sampling interval △ , we obtain Equation 2:

$$\begin{array}{cc} h^{\prime }\left(t\right)=\; & \bar{A}h\left(t\right)+\bar{B}x\left(t\right)\\ y\left(t\right)=\; & Ch\left(t\right) \end{array}$$ (2)

where $\bar{A}=exp\left(△A\right),\bar{B}={\left(△A\right)}^{-1}\left(exp\left(△A\right)-I\right)\cdot △B$ , and △ controls the timescale of the dynamics.

The recurrent update above can be implemented efficiently as a one-dimensional convolution, as shown in Equation 3:

$$\begin{array}{cc} y= & x*\bar{K}\\ \bar{K}= & \left[C\bar{B},C\bar{AB},C{\bar{A}}^{2}\bar{B},\ldots ,C{\bar{A}}^{L-1}\bar{B}\right] \end{array}$$ (3)

where ∗ denotes convolution, $\bar{K}\in R^{L}$ is the convolution kernel induced by the SSM, and L is the length of x . This view allows SSMs to model long-range dependencies in linear time.

The ViM model (Liu Y. et al., 2024) adapts this SSM framework for visual tasks. Its architecture features two core components: the S6 block, which leverages the efficient convolutional representation to model dependencies within a sequence, and the SS2D mechanism, which flattens 2D image features into 1D sequences for the SSM. By employing bidirectional scanning (e.g., horizontal and vertical), SS2D embeds spatial context from the original image grid. Integrated into a U-Net architecture (Ronneberger et al., 2015), ViM has demonstrated strong performance in medical image segmentation. Despite its success, ViM exhibits two fundamental limitations. First, its capacity for fine-grained local modeling is constrained by the inherently 1D nature of the underlying SSM. Second, its fixed, axis-aligned scanning strategy is suboptimal for capturing the complex boundaries and irregular topologies characteristic of anatomical structures. Addressing these shortcomings is the primary motivation for our work. We enhance ViM by introducing mechanisms that significantly boost both local modeling fidelity and scanning flexibility, resulting in the recursion-enhanced ${\text{ZR}}^{2}$ ViM model.

### 3.2 Overall architecture

We introduce ${\text{ZR}}^{2}$ ViM, a recursive visual state space model designed for high-fidelity medical image segmentation. While built upon the classic U-shaped encoder-decoder architecture (Ronneberger et al., 2015), ${\text{ZR}}^{2}$ ViM’s innovation lies in its core building blocks and sequence modeling mechanisms. Central to our design is the novel ${\text{ZR}}^{2}$ Block, which replaces the conventional S6 state space unit. The ${\text{ZR}}^{2}$ Block integrates a new attention mechanism, termed CZ-WKV attention. This block employs SSR within a NRC to model local and global features concurrently. This structure is complemented by an extensible multi-directional zigzag scanning strategy, which, combined with Quad-Directional Token Shift (Q-Shift), injects directional priors into the model. This architecture explicitly enhances spatial continuity, directional robustness, and cross-scale contextual modeling, all while maintaining near-linear computational complexity.

**FIGURE 1:**
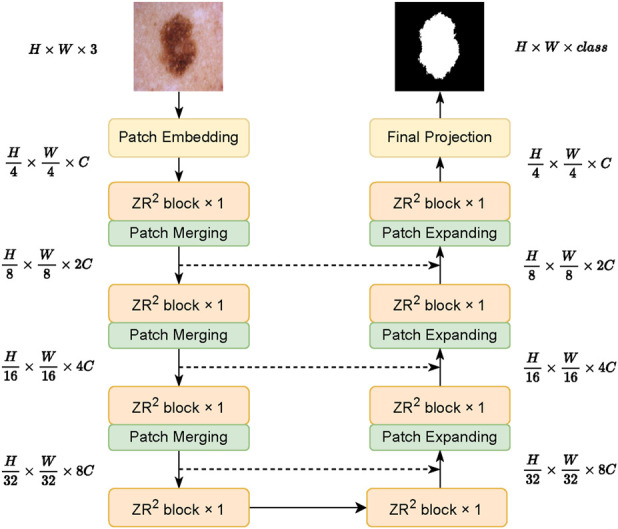
Overall architecture of ${\text{ZR}}^{2}$ ViM. The model employs a U-shaped encoder–decoder framework where the ${\text{ZR}}^{2}$ Block, as the fundamental building block, integrates multi-scale features through skip connections.

The overall architecture of ${\text{ZR}}^{2}$ ViM is illustrated in Figure 1. It consists of a patch-embedding layer, a hierarchical encoder, a symmetric decoder, skip connections, and a final projection layer. An input image $x\in R^{H\times W\times 3}$ is first partitioned into non-overlapping patches using a 4×4 convolution with a stride of 4. This operation simultaneously projects the patches into a C -dimensional feature space, yielding an embedded feature map $x^{\prime }\in R^{\frac{H}{4}\times \frac{W}{4}\times C}$ .

The encoder comprises S=4 stages. Let $x_{i}^{\mathrm{enc}}\in R^{H_{i}\times W_{i}\times C_{i}}$ denote the feature map at the i -th encoder stage. Each stage consists of a series of ${\text{ZR}}^{2}$ Block for feature extraction, followed by a Patch Merging (PM) module (Liu et al., 2021). The PM module downsamples the feature map, halving its spatial resolution $H_{i+1}=H_{i}/2,W_{i+1}=W_{i}/2$ , and doubling its channel dimension $C_{i+1}=2C_{i}$ . The stage-level update for the encoder is thus given in Equation 4:

$$x_{i+1}^{\mathrm{enc}}=PM\left(ZR^{2}\left(x_{i}^{\mathrm{enc}}\right)\right),\;i=1,\ldots ,S-1.$$ (4)

Symmetrical to the encoder, the decoder also comprises S stages. Let $x_{j}^{\mathrm{dec}}\in R^{H_{j}^{\prime }\times W_{j}^{\prime }\times C_{j}^{\prime }}$ be the feature map at the j -th decoder stage. The decoding process begins with the bottleneck feature map from the encoder, $x_{1}^{\mathrm{dec}}=x_{S}^{\mathrm{enc}}$ . Each decoder stage includes several ${\text{ZR}}^{2}$ Block for feature refinement and a Patch Expanding (PE) module (Liu et al., 2021). The PE module upsamples the feature map, doubling its spatial resolution and halving its channel dimension. Skip connections fuse the upsampled decoder features with the corresponding high-resolution features from the encoder. This stage-wise decoder update is defined as in Equation 5:

$$x_{j+1}^{\mathrm{dec}}=ZR^{2}\left(PE\left(x_{i}^{\mathrm{dec}}\right)+x_{S-j}^{\mathrm{enc}}\right),\;j=1,\ldots ,S-1,$$ (5)

where the addition operation fuses the features from the decoder pathway and the corresponding encoder stage.

This symmetric, multi-scale architecture, combined with the recursive feature refinement of the ${\text{ZR}}^{2}$ Block and residual cross-layer fusion, enhances long-range dependency modeling and ensures robust feature consistency across scales.

### 3.3 ZR^2^ block

**FIGURE 2:**
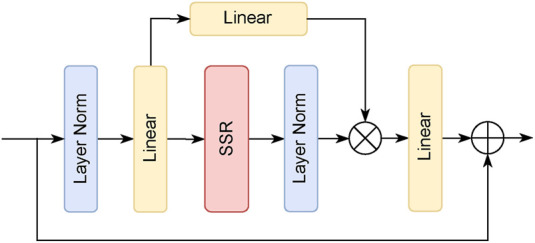
Architecture of the ${\text{ZR}}^{2}$ Block. The ${\text{ZR}}^{2}$ Block, the foundational computational unit of ${\text{ZR}}^{2}$ ViM, comprises a central SSR operator, a normalization layer, a linear projection, and a gated residual connection.

The ${\text{ZR}}^{2}$ Block is the fundamental computational unit of ${\text{ZR}}^{2}$ ViM. It adapts the ViM paradigm of lightweight state space modeling but crucially substitutes the standard S6 operator with our SSR module to explicitly model spatial continuity. As depicted in Figure 2, input features first undergo Layer Normalization and linear projection. The resulting features are then processed by the SSR module, which performs the recursive state updates. The output from the SSR is subsequently normalized, modulated by a sigmoid gate, and fused with the original input via a residual connection to ensure stable information propagation.

The efficacy of this design stems from the SSR module’s unique ability to jointly model fine-grained local details and global context through its nested recursive structure and multi-directional scanning. By integrating this module, the ${\text{ZR}}^{2}$ Block surpasses the performance of the original S6 operator in ViM (Liu Y. et al., 2024), particularly in delineating the complex boundaries and irregular structures characteristic of medical images.

### 3.4 SSR module

#### 3.4.1 NRC collaborative architecture

**FIGURE 3:**
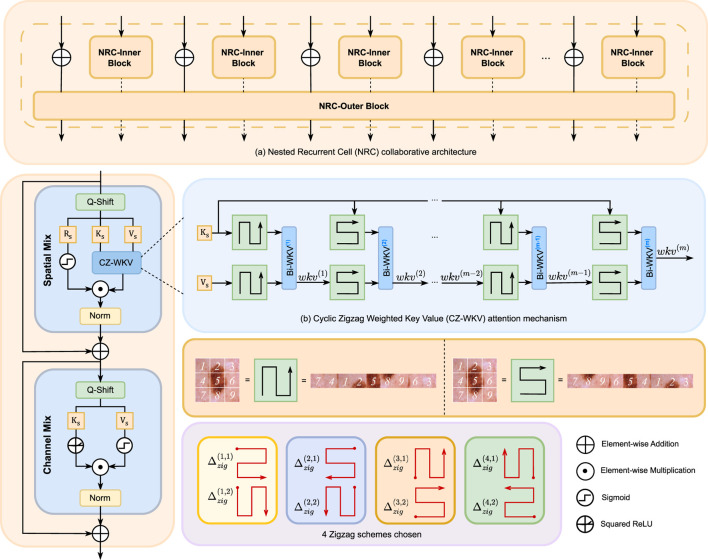
Architecture of the SSR module. **(a)** The NRC structure, which integrates multiple NRC-Inner blocks with a single NRC-Outer block to jointly model local and global spatial relationships. **(b)** The CZ-WKV attention mechanism, which aggregates multi-directional context by recursively applying m Bi-WKV steps across four distinct zigzag scanning patterns.

The architecture of the SSR module is detailed in Figure 3. Its core design features a nested dual-path system composed of an NRC-Inner and an NRC-Outer pathway (Figure 3a). The NRC-Inner path processes fine-grained tokens within local image patches to capture high-frequency details and local spatial dependencies. Concurrently, the NRC-Outer path models the global context across these patches, capturing the long-range dependencies essential for holistic scene understanding. A multi-directional zigzag scanning strategy is applied to both pathways, creating a shared geometric prior that ensures spatial consistency and sequence alignment during their interaction.

In the NRC-Inner path, let k∈{1,…,K} denote the index of the NRC-Inner update (patch group) within the l -th layer. An input token sequence $W_{l,k-1}\in R^{T\times C}$ (where T is the sequence length and C is the channel dimension) undergoes a recursive refinement process. The sequence is first normalized to stabilize the feature distribution. Next, spatial dependencies are modeled using a Spatial Mix operation on the zigzag-ordered sequence, which incorporates a lightweight directional displacement to enhance directional awareness. State updates are then performed via a recursive m -step aggregation based on our CZ-RWKV module. This update is formulated as a residual operation in Equation 6:

$$W_{l,k}=W_{l,k-1}+CZ-RWKV\left(W_{l,k-1}\right),\;k=1,\ldots ,K$$ (6)

here, $W_{l,k}$ represents the refined local-token sequence after the k -th NRC-Inner update in layer l . The CZ-RWKV(⋅) function aggregates information from neighboring tokens along the scan path, allowing the model to progressively refine local details while maintaining representational stability.

The two pathways are coupled via a cross-path feature injection mechanism that allows local features to inform the global context. After its update, the local sequence from the Inner path, $W_{l,k}$ , is summarized into a global descriptor and injected into the Outer path’s state, $S_{l,k-1}$ :

$$S_{l,k-1}^{\prime }=S_{l,k-1}+FC\left(Vec\left(W_{l,k}\right)\right)$$ (7)

in this step, Vec(⋅) flattens the sequence $W_{l,k}$ , and a fully connected layer, FC(⋅) , projects it to match the channel dimension of the NRC-Outer path. This enriched global state is then updated through the Outer path’s own recursive unit, which applies the same CZ-RWKV operator under the shared zigzag scheme:

$$S_{l,k}=S_{l,k-1}^{\prime }+CZ-RWKV\left(LN\left(S_{l,k-1}^{\prime }\right)\right)$$ (8)

here, $S_{l,k}$ denotes the global context representation after the update at step k , and LN(⋅) is layer normalization applied to the previous state. In practice, K is small and the state is updated in-place; we maintain only the current state without storing intermediate $S_{l,k}$ . This cyclical process—where the global memory is first enriched by fine-grained local details (Equation 7) and subsequently updated to model long-range interactions (Equation 8)—enables local structural information to directly inform the global semantic state. This tight integration is critical for preserving spatial continuity along complex object boundaries in medical images.

#### 3.4.2 Quad-directional token shift

The Q-Shift operation (Duan et al., 2024) introduces directional priors before feature serialization to establish local dependencies between adjacent tokens, thereby enabling subsequent multi-directional context modeling. Operationally, for an input feature map $X\in R^{H\times W\times C}$ , Q-Shift splits the channel dimension into four groups. It then shifts each group along one of the four orthogonal directions (up, down, left, right), concatenates the resulting features, and integrates them with the original input X via a gated residual connection, as shown in Equations 9, 10:

$$Q-Shift_{\left(*\right)}\left(X\right)=X+\left(1-\mu_{\left(*\right)}\right)X^{†}$$ (9)

$$\begin{array}{cc} X^{†}\left[h,w\right]=Concat & \left(X\left[h-1,w,0:C/4\right],\right.\\ & X\left[h+1,w,C/4:C/2\right],\\ & X\left[h,w-1,C/2:3C/4\right],\\ & \left.X\left[h,w+1,3C/4:C\right]\right) \end{array}$$ (10)

where $\left(*\right)\in \left\{R,K,V\right\}$ denotes that separate Q-Shift branches are applied for the subsequent computation of R , K , and V , and $\mu_{\left(*\right)}\in R^{C}$ is a learnable, channel-wise gating vector. This quad-directional shifting mechanism explicitly injects anisotropic neighborhood information into distinct channel groups with negligible computational overhead. This process enhances local directional awareness and provides essential guidance for the subsequent CZ-WKV-based recursive modeling.

#### 3.4.3 Spatial mix

Following directional modeling, the feature map is serialized along a zigzag scanning path into a token sequence $X\in R^{T\times C}$ , a process that preserves spatial continuity between adjacent tokens. The Spatial Mix module then processes this sequence as in Equation 11, by first applying the Q-Shift operation and three distinct linear projections to generate the primary components:

$$\begin{array}{cc} R_{s}= & Q-Shift_{R}\left(X\right)W_{R},\\ K_{s}= & Q-Shift_{K}\left(X\right)W_{K},\\ V_{s}= & Q-Shift_{V}\left(X\right)W_{V} \end{array}$$ (11)

where $W_{R},W_{K},W_{V}\in R^{C\times C}$ are learnable projection matrices. Here, $R_{s}$ functions as a content-dependent gating signal, while $K_{s}$ and $V_{s}$ provide the key and value sequences for the subsequent state space computation.

The global spatial response is then computed by the linear-complexity CZ-WKV mechanism as in Equation 12:

$$wkv=CZ-WKV\left(K_{s},V_{s}\right)$$ (12)

this mechanism executes an m -step alternating recursion along multiple zigzag directions, enabling each token to aggregate information from the entire sequence while maintaining spatial ordering. The final output of the Spatial Mix module is formulated as in Equation 13:

$$O_{s}=X+LN\left(\left(\sigma \left(R_{s}\right)⊙wkv\right)W_{O}\right)$$ (13)

where $\sigma \left(\cdot \right)$ is the sigmoid function, $W_{O}\in R^{C\times C}$ is an output projection matrix, ⊙ denotes element-wise multiplication, and LN(⋅) represents Layer Normalization. In this formulation, $\sigma \left(R_{s}\right)$ acts as a dynamic gate, modulating the infusion of the global response wkv into each token. The residual connection with X ensures the preservation of the original local representation. Consequently, the Spatial Mix module effectively integrates long-range spatial context with local feature consistency.

#### 3.4.4 Channel mix

The Channel Mixing module is designed to fuse cross-semantic features along the channel dimension (Zhou et al., 2023), functioning as a gated feed-forward branch that complements the Spatial Mix module. This module operates on the spatially enhanced features $O_{s}$ , again applying the Q-Shift operation and distinct linear projections to generate a gating descriptor and a pre-activation sequence, as shown in Equation 14:

$$\begin{array}{cc} R_{c}=\; & Q-Shift_{R}\left(O_{s}\right)W_{R},\\ K_{c}=\; & Q-Shift_{K}\left(O_{s}\right)W_{K} \end{array}$$ (14)

here, $R_{c}$ functions as a channel-wise gating descriptor, while $K_{c}$ serves as the pre-activation input for constructing the value branch. The value sequence $V_{c}$ is then computed as in Equation 15:

$$V_{c}=SquaredReLU\left(K_{c}\right)W_{V}$$ (15)

where the SquaredReLU(⋅) activation function suppresses negative responses and amplifies strong positive activations. The final output, $O_{c}$ , is computed via a gated residual connection as in Equation 16:

$$O_{c}=O_{s}+LN\left(\left(\sigma \left(R_{c}\right)⊙V_{c}\right)W_{O}\right)$$ (16)

the core of this operation is the element-wise product $\sigma \left(R_{c}\right)⊙V_{c}$ , which functions as a dynamic, data-dependent gate to selectively amplify informative channels while attenuating irrelevant ones. The residual connection with $O_{s}$ preserves the input feature representation, enabling the module to enhance nonlinear cross-channel interactions efficiently and without the quadratic computational complexity typical of self-attention mechanisms.

### 3.5 CZ-WKV attention mechanism

The Bidirectional WKV (Bi-WKV) module, a core component of the Vision-RWKV (Duan et al., 2024) spatial mixture, effectively models long-range dependencies in linear time. However, its performance is highly sensitive to the orientation of token scanning. As the scanning path changes across a 2D image, the resulting token sequence is altered, leading to inconsistent model outputs. While subsequent methods like Re-WKV (Yang et al., 2025) mitigate this sensitivity by applying Bi-WKV across multiple scanning directions, this approach disrupts the image’s inherent spatial continuity. Consequently, it compromises the model’s ability to leverage crucial image-space inductive biases. Zigzag-WKV (Chen et al., 2025) addresses this by preserving spatial continuity via a zigzag scanning pattern. Nevertheless, its single-pass, unidirectional computation provides only a static representation of global context, limiting its capacity to model complex, long-range dependencies effectively. To overcome these limitations, we introduce the CZ-WKV module (Figure 3b). CZ-WKV performs a cascaded, m -step sequence of Bi-WKV operations along a single zigzag path. This design enables the model to dynamically and recursively refine global token interactions. As a result, it preserves the global receptive field and spatial continuity of zigzag scanning while achieving robust performance irrespective of the initial scanning orientation.

#### 3.5.1 Bi-WKV

To address the limited receptive field of the unidirectional WKV (Uni-WKV) mechanism, we adopt the Bi-WKV formulation from Vision-RWKV (Duan et al., 2024). This bidirectional approach expands the receptive field to encompass the entire token sequence, enabling global context modeling while maintaining linear-time complexity. Specifically, for a given projected key $K_{s}$ and value $V_{s}$ , the attention output for the t -th token, $wkv_{t}\in R^{C}$ , is computed as in Equation 17:

$$wkv_{t}=Bi-WKV{\left(K_{s},V_{s}\right)}_{t}=\frac{\sum_{i=1,i\neq t}^{T}e^{-\frac{|t-i|-1}{T}w+k_{i}}v_{i}+e^{u+k_{t}}v_{t}}{\sum_{i=1,i\neq t}^{T}e^{-\frac{|t-i|-1}{T}w+k_{i}}+e^{u+k_{t}}}$$ (17)

in this formulation, T is the total number of tokens, while $k_{i},v_{i}\in R^{C}$ are the key and value vectors for the i -th token, respectively. The term −(|t−i|−1)/T encodes the relative position between tokens t and i . A learnable vector $w\in R^{C}$ modulates the spatial decay based on this relative position, while a second learnable vector, $u\in R^{C}$ , applies a specific weighting to the current token t , thereby amplifying its feature contribution.

The Bi-WKV mechanism concurrently achieves a global receptive field and high computational efficiency. First, the output for each token incorporates information from all other tokens in the sequence, thereby establishing a global receptive field. Second, this mechanism avoids the quadratic complexity characteristic of standard self-attention by eliminating explicit query-key matrix multiplications. For an input sequence of length T with channel dimension C , the computational cost of Bi-WKV scales linearly with T (i.e., O(T×C) ), as demonstrated in Vision-RWKV (Duan et al., 2024). This linear scalability makes the model particularly well-suited for processing the long token sequences generated from high-resolution medical images, where standard attention mechanisms would be computationally prohibitive.

#### 3.5.2 CZ-WKV

Segmenting 2D medical images—such as those from dermatoscopy, ultrasound, or single-slice CT—presents substantial challenges. These images are frequently characterized by low contrast, complex boundaries that are often elongated or jagged, significant geometric deformations, and wide variations in lesion shape and scale. Consequently, an effective segmentation model must satisfy two critical requirements: it must preserve local spatial continuity to honor anatomical and geometric priors, and simultaneously model global, long-range dependencies to achieve robustness against changes in object orientation and scanning direction. Existing state space methods, however, fall short of concurrently meeting these demands. The standard Bi-WKV is sensitive to the sequence unrolling direction. While Re-WKV introduces multi-directional interactions, it does so at the cost of disrupting 2D spatial adjacency. Conversely, Zigzag-WKV maintains spatial continuity but is limited by a fixed scanning path in each forward pass, which provides insufficient cross-layer global context for complex structures. To address these limitations, we propose the CZ-WKV. This module executes a scalable m -step sequence of Bi-WKV operations, where each step is guided by a cyclically shifting zigzag scanning pattern. This design strategically balances directional robustness with the preservation of spatial continuity, all while maintaining linear-time efficiency. The core of CZ-WKV is the following recursive formulation:

$$wkv^{\left(j\right)}=Bi-WKV^{\left(j\right)}\left(P_{\mathrm{zig}}^{\left(i,j\right)}\left(K_{s}\right),P_{\mathrm{zig}}^{\left(i,j\right)}\left(wkv^{\left(j-1\right)}\right)\right)$$ (18)

let $Bi-WKV^{\left(j\right)}\left(\cdot \right)$ denote the j -th Bi-WKV operation and $P_{\mathrm{zig}}^{\left(i,j\right)}\left(\cdot \right)$ represent the spatial permutation operation corresponding to the scanning direction for the i -th zigzag scheme at step j , where i∈{1,2,3,4} and j∈{1,…,m} . Within a given scheme i , the scanning direction, defined by $P_{\mathrm{zig}}$ , alternates between successive steps (e.g., $P_{\mathrm{zig}}^{\left(i,1\right)}$ for odd steps, $P_{\mathrm{zig}}^{\left(i,2\right)}$ for even steps) to ensure comprehensive feature aggregation from opposing orientations. To promote directional diversity across layers, the scheme index is cycled as $s_{l}=1+\left(l\;mod\;4\right)$ , which traverses the four distinct zigzag patterns in a round-robin manner.

The process is defined recursively. We denote $wkv^{\left(j\right)}$ as the attention output of the j -th Bi-WKV iteration. The process is initialized using the value projection $V_{s}$ , such that $wkv^{\left(0\right)}=V_{s}$ . As formulated in Equation 18, at each subsequent step j , the Bi-WKV module uses the output from the preceding iteration, $wkv^{\left(j-1\right)}$ , as its new value input. This recursive mechanism thereby integrates the attention output derived from a different scanning direction in the previous step. After m iterations, the final output is given by Equation 19:

$$wkv=CZ-WKV\left(K_{s},V_{s}\right)=wkv^{\left(m\right)}$$ (19)

Thus, CZ-WKV synergistically combines a recurrent attention mechanism with multiple, cycling zigzag scanning paths. This approach strengthens global token interactions far more effectively than Zigzag-WKV while, unlike Re-WKV, preserving vital 2D spatial continuity. Furthermore, this enhanced modeling capability is achieved without sacrificing computational efficiency. As the number of iterations m is a small constant much less than the sequence length m≪T , the computational complexity remains linear with respect to sequence length, scaling as O(TC) . This makes the proposed CZ-WKV module an efficient yet powerful solution for robust medical image segmentation.

### 3.6 Loss function

To train our model, we employ a composite loss function engineered to balance pixel-level precision with region-level consistency. This function is the sum of a cross-entropy (CE) loss and a Dice loss, formulated as in Equation 20:

$$L=L_{CE}+L_{\mathrm{Dice}}$$ (20)

the CE component, $L_{CE}$ , targets pixel-wise classification accuracy, while the Dice component, $L_{\mathrm{Dice}}$ , addresses the common challenge of class imbalance in medical segmentation by maximizing the geometric overlap between the model’s prediction and the ground-truth annotation (Isensee et al., 2021; Wu et al., 2024). The cross-entropy loss is defined as in Equation 21:

$$L_{CE}=-\frac{1}{N}\overset{N}{\underset{i=1}{\sum }}\overset{C}{\underset{c=1}{\sum }}y_{i,c}\log\left({\hat{y}}_{i,c}\right)$$ (21)

and the Dice loss is defined as in Equation 22:

$$L_{\mathrm{Dice}}=1-\frac{2\sum_{i=1}^{N}y_{i}{\hat{y}}_{i}+1}{\sum_{i=1}^{N}y_{i}+\sum_{i=1}^{N}{\hat{y}}_{i}+1}$$ (22)

In these equations, N is the total number of pixels in a batch and C is the number of segmentation classes. For a given pixel i , $y_{i,c}$ is a binary indicator for the ground-truth label (1 if pixel i belongs to class c ; 0 otherwise), and ${\hat{y}}_{i,c}$ is the model’s predicted probability of pixel i belonging to class c . The terms $y_{i}$ and ${\hat{y}}_{i}$ represent the flattened ground-truth and prediction vectors, respectively. This dual-component loss function compels the model to produce segmentations that are not only precise at the pixel level but also structurally coherent, which is critical for delineating fine anatomical details and ensuring region integrity.

## 4 Experiments

### 4.1 Datasets

To assess the performance and scalability of ${\text{ZR}}^{2}$ ViM, we benchmarked the model on five publicly available medical image segmentation datasets. These datasets encompass a range of clinical applications—skin, breast lesion, colorectal polyp, and organ segmentation—and feature diverse imaging modalities and resolutions, providing a comprehensive testbed for evaluating the model’s efficacy and generalizability.

1. Skin lesion datasets. We utilized two benchmarks from the International Skin Imaging Consortium (ISIC): ISIC 2017 (Berseth, 2017) and ISIC 2018 (Codella et al., 2019). The ISIC 2017 dataset contains 2,150 images and ISIC 2018 contains 2,694 images; each image is paired with a corresponding ground-truth lesion mask. Following established protocols (Ruan et al., 2024), we partitioned ISIC 2017 into training (1,500 images) and test (650 images) sets. For ISIC 2018, the split was 1,886 images for training and 808 for testing.
2. Polyp dataset. For polyp segmentation, we used the CVC-ClinicDB dataset (Bernal et al., 2015), originally from the MICCAI 2015 colonoscopic polyp detection challenge. This dataset comprises 612 colonoscopic images with expert-annotated polyp masks. It presents clinically relevant challenges, including polyps of varying sizes and morphologies, inconsistent illumination, complex mucosal structures, and specular artifacts. Adhering to standard splits (Jha et al., 2019), we divided the dataset into 429 images for training and 183 for testing.
3. Ultrasound dataset. The Breast Ultrasound Images (BUSI) dataset (Al-Dhabyani et al., 2020) was used to evaluate performance on ultrasound data. It consists of 780 images, each with a ground-truth mask of a breast lesion. This dataset is particularly challenging due to inherent speckle noise, low contrast, and the irregular lesion morphologies characteristic of ultrasound imaging. The dataset was partitioned into 624 training and 156 test images.
4. Multi-organ dataset. To evaluate multi-organ segmentation, we employed the Synapse multi-organ CT dataset from the MICCAI 2015 Multi-Atlas Abdomen Labeling Challenge (Landman et al., 2015). This dataset includes 30 abdominal CT volumes, corresponding to 3,779 axial slices, with segmentations for eight organs: aorta, gallbladder, left kidney, right kidney, liver, pancreas, spleen, and stomach. Consistent with prior work, we used 18 cases for training.

### 4.2 Implementation details

We applied a standardized training protocol across all segmentation tasks to ensure fair and reproducible comparisons. All models were trained and evaluated using the PyTorch framework on a single NVIDIA RTX 3080 GPU. Input images were uniformly resized to 256×256 pixels. To prevent overfitting and enhance model generalizability, we applied online data augmentation, including random horizontal flips, vertical flips, and rotations. Models were trained for 150 epochs with a batch size of 16 using the AdamW optimizer (Zhou et al., 2024). The learning rate was initialized to 1e-3 and adjusted using a linear warmup schedule followed by polynomial decay.

Following common practice, we further split 10% of the training set as a validation set for model selection and checkpointing, while keeping the official test split unchanged and using it only for final evaluation. The validation split was fixed across random seeds to ensure fair paired comparisons. Unless otherwise specified, all reported metrics are obtained using the checkpoint with the best validation DSC on each run. To account for training variability, all experiments were repeated five times using different random seeds while keeping the data splits and all hyperparameters fixed. For fair paired comparisons, the same set of seeds was used for all competing methods on each benchmark.

**FIGURE 4:**
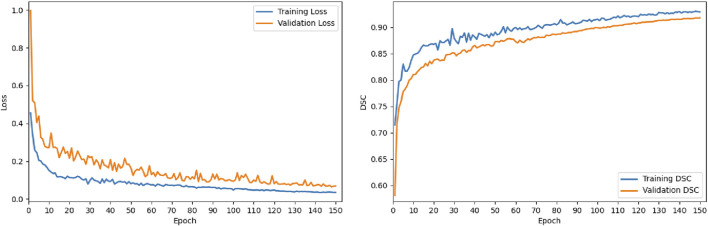
Training and validation curves on ISIC 2018. The plots show the loss (left) and DSC (right) over 150 epochs, indicating stable convergence.

The results reported in Tables 1–4 are presented as mean ± standard deviation over the five runs. To further demonstrate optimization stability and convergence behavior under this standardized protocol, we provide training and validation curves on ISIC 2018 in Figure 4, including loss and Dice Similarity Coefficient (DSC) over 150 epochs. For visual clarity, we plot one run for illustration.

**TABLE 1:** Performance comparison with state-of-the-art methods on the ISIC 2017 dataset.

Type	Method	DSC↑	mIoU↑	Acc↑	Spe↑	Sen↑	BFS↑
CNN	UNet (Ronneberger et al., 2015)	86.99 ± 0.89	76.98 ± 0.50	94.65 ± 0.49	97.43 ± 0.12	86.82 ± 0.94	83.81 ± 0.55
	UNet++ (Zhou et al., 2018)	86.00 ± 0.82	75.44 ± 0.62	94.35 ± 0.47	97.34 ± 0.11	85.40 ± 0.91	83.42 ± 0.43
	Att-Unet (Oktay et al., 2018)	87.08 ± 1.18	77.12 ± 0.53	94.79 ± 0.52	97.78 ± 0.13	85.65 ± 1.20	84.12 ± 0.78
Transformer	TransUNet (Chen et al., 2024)	88.13 ± 0.78	78.79 ± 0.57	95.12 ± 0.41	98.14 ± 0.11	86.05 ± 1.01	85.47 ± 0.47
	TransFuse (Zhang et al., 2021)	84.40 ± 0.71	79.21 ± 0.45	95.17 ± 0.39	97.98 ± 0.14	87.14 ± 0.64	82.31 ± 0.29
	TC-Net (Dong et al., 2022)	87.23 ± 0.91	77.35 ± 0.43	94.84 ± 0.58	98.05 ± 1.16	85.85 ± 0.81	84.28 ± 0.48
SSM	VM-UNet (Ruan et al., 2024)	89.03 ± 0.96	80.23 ± 0.40	95.29 ± 0.42	97.58 ± 0.10	**89.90** ± **0.15**	86.51 ± 0.58
	CC-ViM (Zhu et al., 2025)	89.74 ± 0.91	81.40 ± 0.38	95.60 ± 0.49	98.19 ± 0.90	88.70 ± 0.95	86.72 ± 0.55
	SliceMamba (Fan et al., 2025)	89.93 ± 0.71	81.70 ± 0.36	**95.75** ± **0.56**	98.30 ± 1.06	88.81 ± 0.93	86.98 ± 0.35
	SA-UMamba (Liu et al., 2025)	89.40 ± 0.93	80.83 ± 0.34	94.44 ± 0.53	97.82 ± 0.15	89.60 ± 0.87	86.32 ± 0.55
RWKV	Zig-RiR (Chen et al., 2025)	84.71 ± 0.76	76.76 ± 0.42	95.10 ± 1.27	98.21 ± 1.68	88.64 ± 1.28	82.56 ± 0.34
	HER-Seg (Xu et al., 2025)	87.71 ± 0.64	80.85 ± 0.44	94.76 ± 1.65	97.83 ± 0.66	88.26 ± 0.98	84.38 ± 0.46
	ZR^2^ViM (Ours)	**92.12** ± **0.27**	**85.83** ± **0.31**	95.68 ± 0.34	**98.36** ± **0.12**	89.86 ± 0.91	**89.64** ± **0.23**

Top results are highlighted in bold. Results are mean ± SD over five runs with different random seeds. BFS uses a 2-pixel tolerance at 256×256 with the same protocol for all methods.

**TABLE 2:** Performance comparison with state-of-the-art methods on the ISIC 2018 dataset.

Type	Method	DSC↑	mIoU↑	Acc↑	Spe↑	Sen↑	BFS↑
CNN	UNet (Ronneberger et al., 2015)	87.55 ± 1.01	77.86 ± 0.58	93.05 ± 0.37	96.69 ± 0.62	85.86 ± 0.48	84.52 ± 0.93
	UNet++ (Zhou et al., 2018)	87.83 ± 0.79	78.31 ± 0.61	93.02 ± 0.46	95.75 ± 0.49	88.65 ± 0.93	85.23 ± 0.77
	Att-Unet (Oktay et al., 2018)	87.91 ± 0.74	78.43 ± 0.49	93.13 ± 0.30	96.23 ± 0.33	87.60 ± 0.89	84.89 ± 0.75
Transformer	TransUNet (Chen et al., 2024)	89.56 ± 0.42	81.09 ± 0.51	93.99 ± 0.24	97.02 ± 0.33	88.14 ± 0.49	86.78 ± 0.62
	TransFuse (Zhang et al., 2021)	89.27 ± 0.35	80.63 ± 0.47	93.66 ± 0.23	95.74 ± 0.28	**91.26** ± **0.67**	87.12 ± 0.81
	TC-Net (Dong et al., 2022)	88.25 ± 0.89	78.97 ± 0.44	93.32 ± 0.55	96.48 ± 0.57	87.60 ± 0.52	85.31 ± 0.73
SSM	VM-UNet (Ruan et al., 2024)	89.71 ± 0.78	81.35 ± 0.42	93.91 ± 0.25	96.13 ± 0.14	91.12 ± 0.31	87.36 ± 0.66
	CC-ViM (Zhu et al., 2025)	90.06 ± 0.56	81.92 ± 0.39	94.23 ± 0.39	**97.32** ± **0.57**	88.74 ± 0.40	87.42 ± 0.38
	SliceMamba (Fan et al., 2025)	90.30 ± 0.09	82.32 ± 0.36	94.29 ± 0.54	97.14 ± 0.59	89.58 ± 0.82	87.89 ± 0.95
	SA-UMamba (Liu et al., 2025)	89.49 ± 0.71	80.98 ± 0.40	85.90 ± 0.35	96.75 ± 0.42	89.16 ± 0.90	86.81 ± 0.55
RWKV	Zig-RiR (Chen et al., 2025)	87.42 ± 0.13	79.78 ± 0.49	94.02 ± 0.53	95.18 ± 0.98	89.50 ± 0.25	85.05 ± 0.35
	HER-Seg (Xu et al., 2025)	88.63 ± 0.17	81.62 ± 0.46	93.74 ± 0.47	95.75 ± 0.87	88.65 ± 0.38	86.11 ± 0.28
	ZR^2^ViM (Ours)	**92.22** ± **0.41**	**85.65** ± **0.34**	**94.33** ± **0.26**	97.24 ± 0.23	91.18 ± 0.80	**90.25** ± **0.21**

Top results are highlighted in bold. Results are mean ± SD over five runs with different random seeds. BFS uses a 2-pixel tolerance at 256×256 with the same protocol for all methods.

**TABLE 3:** Performance comparison with state-of-the-art methods on the BUSI and CVC-ClinicDB datasets.

Type	Method	BUSI			CVC-ClinicDB		
		DSC ↑	mIoU ↑	BFS ↑	DSC ↑	mIoU ↑	BFS ↑
CNN	UNet (Ronneberger et al., 2015)	76.33 ± 0.60	62.40 ± 0.53	63.48 ± 1.28	82.72 ± 0.72	70.53 ± 0.75	74.22 ± 0.16
	UNet++ (Zhou et al., 2018)	76.47 ± 0.89	65.92 ± 0.56	65.62 ± 1.33	81.20 ± 0.53	68.35 ± 0.33	72.70 ± 0.13
	Att-Unet (Oktay et al., 2018)	76.35 ± 0.96	68.46 ± 1.39	65.14 ± 0.96	88.55 ± 0.29	79.46 ± 0.46	81.03 ± 0.05
Transformer	TransUNet (Chen et al., 2024)	71.27 ± 0.86	60.09 ± 0.43	60.27 ± 1.41	86.77 ± 0.40	79.95 ± 0.65	80.42 ± 0.03
	Swin-Unet (Cao et al., 2022)	82.35 ± 0.80	73.65 ± 0.68	72.23 ± 0.88	87.03 ± 0.31	81.68 ± 0.50	81.54 ± 0.21
	MISSFormer (Huang et al., 2022)	75.81 ± 0.92	65.33 ± 0.49	63.81 ± 0.73	86.66 ± 0.48	80.40 ± 0.27	80.16 ± 0.13
SSM	VM-UNet (Ruan et al., 2024)	78.88 ± 0.69	67.55 ± 0.33	66.88 ± 0.85	88.60 ± 0.25	80.50 ± 0.76	82.12 ± 0.18
	CC-ViM (Zhu et al., 2025)	81.39 ± 0.52	73.58 ± 0.59	71.58 ± 0.69	87.73 ± 0.19	81.16 ± 0.35	81.23 ± 0.11
	AEMMamba (Dong et al., 2025)	84.24 ± 0.81	76.12 ± 0.67	74.24 ± 1.14	92.41 ± 0.23	87.69 ± 0.37	86.91 ± 0.07
	Swin-U Mamba (Liu J. et al., 2024)	82.56 ± 0.78	73.62 ± 0.50	72.56 ± 1.01	89.49 ± 0.12	84.47 ± 0.14	84.69 ± 0.05
RWKV	Zig-RiR (Chen et al., 2025)	72.61 ± 0.39	62.55 ± 0.43	61.61 ± 0.95	83.52 ± 0.17	76.22 ± 0.19	77.04 ± 0.08
	HER-Seg (Xu et al., 2025)	70.63 ± 0.67	60.47 ± 0.32	60.79 ± 1.46	87.07 ± 0.15	81.39 ± 0.20	81.57 ± 0.07
	ZR^2^ViM (Ours)	**86.45** ± **0.32**	**77.84** ± **0.24**	**77.11** ± **0.66**	**93.95** ± **0.13**	**89.00** ± **0.18**	**88.68** ± **0.05**

Top results are highlighted in bold. Results are mean ± SD over five runs with different random seeds. BFS uses a 2-pixel tolerance at 256×256 with the same protocol for all methods.

**TABLE 4:** Performance comparison with state-of-the-art methods on the Synapse multi-organ CT dataset.

Type	Method	DSC↑	HD95↓	Aorta	Gallbladder	Kidney(L)	Kidney(R)	Liver	Pancreas	Spleen	Stomach
CNN	UNet (Ronneberger et al., 2015)	76.85 ± 1.19	39.70 ± 0.69	80.07	69.72	77.77	68.60	93.43	53.98	86.67	75.58
	UNet++ (Zhou et al., 2018)	78.11 ± 1.07	36.87 ± 0.42	81.46	68.57	80.46	78.59	93.74	56.94	87.47	77.61
	Att-Unet (Oktay et al., 2018)	77.77 ± 0.86	36.02 ± 0.60	**89.55**	68.88	77.98	71.11	93.57	58.04	87.30	75.75
Transformer	TransUNet (Chen et al., 2024)	77.48 ± 0.91	31.69 ± 1.01	87.23	63.13	81.87	77.02	94.08	55.86	85.08	75.62
	Swin-Unet (Cao et al., 2022)	79.51 ± 0.73	21.55 ± 0.70	85.47	66.53	83.28	79.61	94.29	56.58	90.66	79.62
	MISSFormer (Huang et al., 2022)	81.96 ± 0.85	18.20 ± 0.59	86.99	68.65	85.21	82.00	94.41	65.67	91.92	80.81
SSM	VM-UNet (Ruan et al., 2024)	81.08 ± 0.80	19.21 ± 0.42	86.45	69.49	86.36	82.76	94.17	59.36	89.51	80.54
	CC-ViM (Zhu et al., 2025)	82.65 ± 0.75	17.83 ± 0.47	87.63	68.45	86.23	83.22	94.67	**67.12**	92.05	**81.82**
	SliceMamba (Fan et al., 2025)	81.95 ± 0.77	16.04 ± 0.38	87.78	68.77	88.30	84.26	95.25	64.49	86.91	79.82
	SA-UMamba (Liu et al., 2025)	82.54 ± 0.90	16.80 ± 0.53	88.07	70.46	86.46	83.96	94.42	65.32	89.89	81.76
RWKV	Zig-RiR (Chen et al., 2025)	82.26 ± 0.99	16.65 ± 0.81	88.14	70.15	87.51	83.38	94.29	66.02	90.72	77.86
	HER-Seg (Xu et al., 2025)	82.37 ± 0.64	18.74 ± 0.48	87.46	**71.33**	87.46	84.17	94.75	66.13	89.08	78.59
	ZR^2^ViM (Ours)	**83.04** ± **0.84**	**15.68** ± **0.36**	87.92	69.37	**88.65**	**84.54**	**95.28**	64.42	**92.81**	81.32

Top results are highlighted in bold. Overall DSC and HD95 are reported as mean ± SD over five runs with different random seeds. For space constraints, per-organ results report only the mean DSC over five runs (SD omitted).

### 4.3 Evaluation metrics

To ensure a rigorous evaluation of model generalization across diverse imaging modalities and anatomical structures, we employed task-specific protocols aligned with established benchmarks in medical image segmentation (Isensee et al., 2021; Chen et al., 2024). For 2D segmentation tasks, we quantified performance using a comprehensive suite of metrics, including mean Intersection over Union (mIoU), Dice Similarity Coefficient (DSC), sensitivity (Sen), specificity (Spe), accuracy (Acc), and Boundary F1-score (BFS). While all metrics were computed, we prioritized DSC and mIoU as the primary overlap-based metrics for all 2D benchmarks. To explicitly assess contour accuracy and boundary integrity, we additionally adopted BFS to quantify boundary alignment and contour continuity between predicted masks and ground-truth annotations. For the 3D multi-organ segmentation on the Synapse multi-organ CT dataset, we used DSC to assess volumetric overlap and supplemented it with the 95% Hausdorff distance (HD95) to specifically evaluate the accuracy of boundary delineation. BFS was computed on binarized masks with a boundary tolerance of 2 pixels at 256×256 resolution for all 2D datasets, and the same setting was applied consistently to all methods. We used a standard distance-transform based implementation to match predicted and ground-truth boundaries within the tolerance band (Perazzi et al., 2016).

To account for training variability, all results are reported as mean ± standard deviation (SD) over five independent runs with different random seeds under identical settings. We further computed 95% confidence intervals (CI) of the mean using a t-distribution-based interval across the five runs. CIs are reported for the primary metrics only (DSC/mIoU for 2D tasks and DSC/HD95 for Synapse). For 2D benchmarks, we additionally report the CI for BFS to directly characterize the reliability of boundary accuracy improvements. Statistical significance was evaluated using a two-sided paired t-test on the primary metric between ${\text{ZR}}^{2}$ ViM and the strongest competing baseline on each benchmark, based on the five paired runs with matched random seeds, with a significance level of p<0.05 . The strongest baseline was selected per benchmark according to the primary metric (DSC/mIoU for 2D tasks and DSC/HD95 for Synapse).

These metrics are defined as follows in Equations 23–28:

$$mIoU=\frac{1}{C}\overset{C}{\underset{c=1}{\sum }}\frac{TP_{c}}{TP_{c}+FP_{c}+FN_{c}}$$ (23)

$$DSC=\frac{2TP}{2TP+FP+FN}$$ (24)

$$Acc=\frac{TP+TN}{TP+TN+FP+FN}$$ (25)

$$Sen=\frac{TP}{TP+FN}$$ (26)

$$Spe=\frac{TN}{TN+FP}$$ (27)

$$BFS=\frac{2\cdot P_{b}\cdot R_{b}}{P_{b}+R_{b}}$$ (28)

where TP,FP,TN and FN denote the numbers of true positives, false positives, true negatives, and false negatives, respectively. For mIoU, $TP_{c},FP_{c}$ and $FN_{c}$ are the corresponding values for class c across C total classes. In the BFS equation, $P_{b}$ and $R_{b}$ represent boundary precision and boundary recall, respectively, which are calculated based on the distance between the predicted boundaries and the ground-truth boundaries within a specified tolerance. HD95 is calculated as the 95th percentile of the bidirectional surface distances between the predicted and ground-truth segmentation boundaries.

### 4.4 Comparisons with state-of-the-art methods

We rigorously evaluated the performance and generalization of ${\text{ZR}}^{2}$ ViM across five representative medical image segmentation benchmarks. To ensure a fair and robust comparison, all experiments adhered to identical data splits and standardized evaluation protocols. Across these benchmarks, ${\text{ZR}}^{2}$ ViM consistently achieved superior performance with improved boundary delineation. Improvements on the primary metrics are statistically significant under a two-sided paired t-test (p<0.05) across five matched-seed runs. We further report 95% confidence intervals (CI) of the mean for the primary metrics; the CIs show limited dispersion across seeds, supporting that the observed gains are stable and not driven by random-seed listvariability.

**FIGURE 5:**
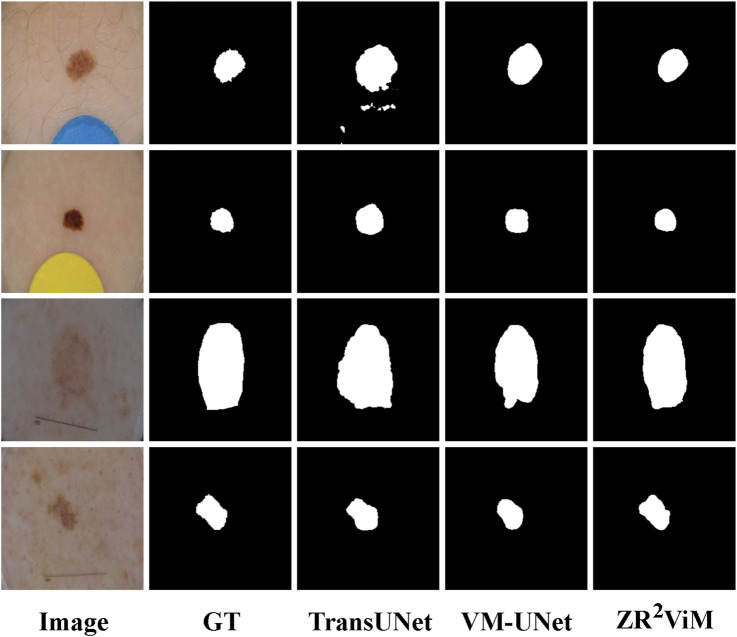
Qualitative segmentation results on the ISIC 2017 and ISIC 2018 datasets.

**FIGURE 6:**
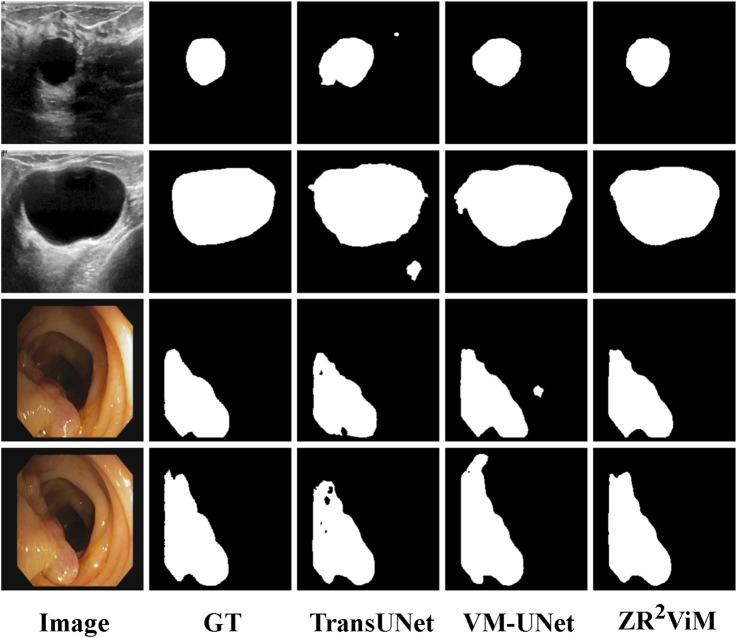
Qualitative segmentation results on the BUSI and CVC-ClinicDB datasets.

**FIGURE 7:**
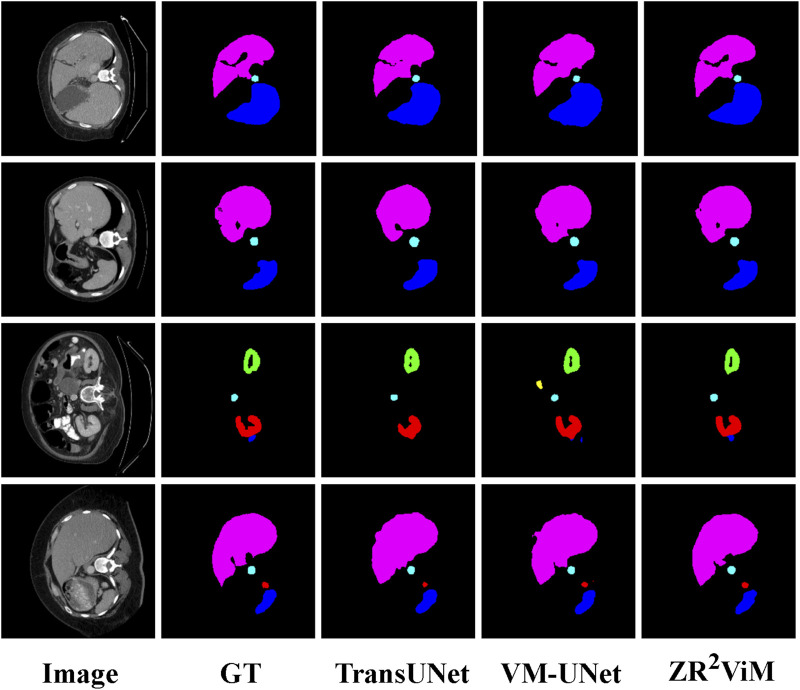
Qualitative segmentation results on the Synapse multi-organ CT dataset.

1. Results on skin lesion segmentation. ZR^2^ViM was evaluated on the ISIC 2017 (Berseth, 2017) and ISIC 2018 (Codella et al., 2019) skin lesion segmentation benchmarks. On these tasks, SSMs such as VM-UNet (Ruan et al., 2024) and CC-ViM (Zhu et al., 2025) generally surpass conventional CNN-based (e.g., U-Net (Ronneberger et al., 2015)) and Transformer-based architectures (e.g., TransUNet (Chen et al., 2024)), largely due to their superior capacity for modeling long-range dependencies. This trend highlights the promise of SSMs for vision tasks. However, ${\text{ZR}}^{2}$ ViM substantially improves upon existing SSMs, establishing new state-of-the-art performance on both benchmarks (Tables 1, 2). On the ISIC 2017 dataset, ${\text{ZR}}^{2}$ ViM achieved a DSC of 92.12% (95% CI: 91.78%–92.46%) and an mIoU of 85.83% (95% CI: 85.45%–86.21%), surpassing the previous leading model, SliceMamba (Fan et al., 2025), by 2.19 and 4.13 percentage points, respectively (DSC: p<0.01 ). Notably, it also attained the highest specificity (98.36%), indicating a low false-positive rate, and achieved a BFS of 89.64% (95% CI: 89.35%–89.93%), reflecting improved boundary alignment. This superior performance was replicated on the ISIC 2018 dataset, where ${\text{ZR}}^{2}$ ViM again outperformed all baseline models, achieving a DSC of 92.22% (95% CI: 91.71%–92.73%) and an mIoU of 85.65% (95% CI: 85.23%–86.07%) (improvements of 1.92 and 3.33 percentage points over SliceMamba, DSC: p<0.01 ). Furthermore, the model achieved a BFS of 90.25% (95% CI: 89.99%–90.51%), quantitatively confirming the finer margin delineation observed in the qualitative results (Figure 5). Qualitative results (Figure 5) visually corroborate these quantitative gains. Predictions from ${\text{ZR}}^{2}$ ViM align more closely with ground-truth contours, producing segmentation masks with more continuous boundaries and fewer false positives or extensions. This precision is particularly evident for lesions with intricate boundaries, irregular morphologies, and small sizes. In contrast, models like TransUNet (Chen et al., 2024) and VM-UNet (Ruan et al., 2024) frequently produce over-segmented or blurred boundaries. These improvements stem directly from the architectural innovations of ${\text{ZR}}^{2}$ ViM. The scalable, multi-directional zigzag scanning ensures that the model captures features with directional robustness, which is critical for preserving boundary continuity. Concurrently, the nested recursive connections within the SSR module enable an efficient fusion of fine-grained local details with global contextual information. This dual-pronged approach allows ${\text{ZR}}^{2}$ ViM to excel at characterizing fine local features without sacrificing global region consistency, leading to more accurate and reliable segmentation.
2. Results on breast ultrasound and colorectal polyp segmentation To assess its generalization capabilities, we evaluated ${\text{ZR}}^{2}$ ViM on two clinically challenging and distinct imaging modalities: breast ultrasound (BUSI (Al-Dhabyani et al., 2020)) and colonoscopic polyp imaging (CVC-ClinicDB (Bernal et al., 2015)). These datasets present formidable challenges, including segmenting lesions obscured by severe speckle noise in ultrasound and delineating polyps with varied morphologies against low-contrast mucosa in endoscopy. On the BUSI dataset, ${\text{ZR}}^{2}$ ViM established state-of-the-art performance, achieving a DSC of 86.45% (95% CI: 86.05%–86.85%) and an mIoU of 77.84% (95% CI: 77.54%–78.14%) (Table 3). This represents a significant (p<0.05) 2.21 percentage point improvement in DSC over the next-best model, AEMMamba (Dong et al., 2025). Notably, the high BFS of 77.11% (95% CI: 76.29%–77.93%) demonstrates the model’s capability to accurately localize lesion boundaries even in the presence of severe speckle noise. This performance gain is directly attributable to the multi-directional recurrent aggregation within the CZ-WKV module, which effectively suppresses acoustic artifacts and enhances boundary discrimination in low-contrast conditions. Similarly, on the CVC-ClinicDB dataset, ${\text{ZR}}^{2}$ ViM again surpassed all baselines, achieving a DSC of 93.95% (95% CI: 93.79%–94.11%) and an mIoU of 89.00% (95% CI: 88.78%–89.22%). This result outperformed AEMMamba (Dong et al., 2025) by 1.54 and 1.31 percentage points, respectively (DSC: p<0.05 ). The model also achieved a robust BFS of 88.68% (95% CI: 88.62%–88.74%), substantiating its effectiveness in delineating polyp margins against low-contrast mucosa. To our knowledge, a DSC of 93.95% is unprecedented for this benchmark, establishing a new state-of-the-art performance. Qualitative analysis further substantiates these quantitative results (Figure 6). In breast ultrasound images, ${\text{ZR}}^{2}$ ViM demonstrates a remarkable ability to suppress speckle noise while accurately localizing lesion boundaries. In colonoscopic images, it produces visibly finer margin delineation and superior structural continuity compared to competing methods. Collectively, these results across two disparate and challenging modalities underscore the robustness and broad applicability of the ${\text{ZR}}^{2}$ ViM architecture.
3. Results on synapse multi-organ CT segmentation. We further assessed ${\text{ZR}}^{2}$ ViM on the Synapse multi-organ CT dataset (Landman et al., 2015), a demanding benchmark characterized by intricate anatomical structures, significant scale variation, and complex inter-organ boundaries. This task places stringent requirements on a model’s ability to integrate global context with fine-grained local detail. ${\text{ZR}}^{2}$ ViM established new state-of-the-art performance, achieving an average DSC of 83.04% (95% CI: 82.00%–84.08%) (Table 4). More critically, it demonstrated a substantial improvement in boundary delineation accuracy, reducing the average HD95 from 17.83 mm (CC-ViM (Zhu et al., 2025)) to 15.68 mm (95% CI: 15.23–16.13). This represents a clinically significant 12.1% reduction in surface distance error (HD95: p<0.01 ), highlighting the model’s superior precision. An organ-level analysis reveals a balanced and robust performance profile. The model excelled in segmenting large organs, attaining high DSC scores for the liver (95.28%) and left kidney (88.65%), as well as for complex structures like the spleen (92.81%) and aorta (87.92%). While its performance on smaller, more challenging organs like the gallbladder and pancreas was highly competitive, it did not uniformly surpass every baseline. Nevertheless, the absence of pronounced weaknesses on any single organ underscores the model’s reliability for comprehensive anatomical segmentation. Visual inspection of the segmentation results (Figure 7) corroborates these quantitative findings. ${\text{ZR}}^{2}$ ViM consistently generates masks with sharper, more anatomically plausible organ boundaries and superior structural integrity, especially in regions with low tissue contrast. This qualitative evidence directly supports the marked improvement observed in the HD95 metric, confirming the model’s advanced capability for precise 3D segmentation.

Experimental results across five diverse medical imaging datasets establish that ${\text{ZR}}^{2}$ ViM consistently delivers state-of-the-art or near state-of-the-art segmentation performance. Unlike traditional CNNs and Transformers, ${\text{ZR}}^{2}$ ViM maintains the linear-time complexity of SSMs, enabling the efficient modeling of global dependencies. Furthermore, it surpasses other SSM- and RWKV-based counterparts by capturing richer feature representations and achieving superior spatial-modeling accuracy. This enhanced performance is directly attributable to its core architectural innovations: the ${\text{ZR}}^{2}$ Block for nested recursive state modeling, integrated Q-Shift for directional-prior injection, and a scalable multi-directional zigzag scanning mechanism coupled with CZ-WKV attention. Collectively, these innovations render ${\text{ZR}}^{2}$ ViM highly effective for addressing critical challenges in medical imaging, such as segmenting complex boundaries, small targets, and multi-scale structures across various modalities. This combination of high accuracy and computational efficiency positions ${\text{ZR}}^{2}$ ViM as a robust and versatile solution for practical clinical applications.

### 4.5 Efficiency analysis

**FIGURE 8:**
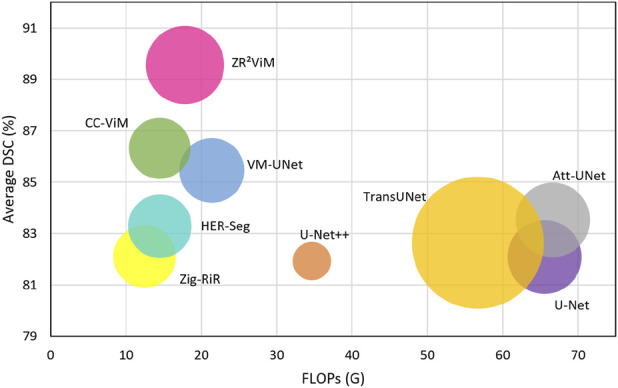
Comparative analysis of model accuracy and efficiency. Each point represents a model, plotting its average DSC coefficient against computational cost (FLOPs) across five medical imaging datasets: ISIC 2017, ISIC 2018, BUSI, CVC-ClinicDB, and Synapse. Bubble size corresponds to the parameter count (M), illustrating the three-way trade-off between performance, computational demand, and model size.

To assess computational efficiency, we evaluate each model in terms of parameter count (Params), floating-point operations (FLOPs), and representative inference latency. For complexity profiling, all methods are measured with a unified input size of 1×3×256×256 using the same profiling script. For efficiency benchmarking in Table 5, all methods are profiled under the same GPU, input resolution, and batch size. Under this setting, ${\text{ZR}}^{2}$ ViM requires 38.66 M parameters and 17.84G FLOPs, positioning it within the lightweight-to-midrange complexity regime. This computational profile places ${\text{ZR}}^{2}$ ViM in a highly advantageous position regarding the accuracy-efficiency trade-off, as illustrated in Figure 8 and detailed in Table 5. The bubble plot (Figure 8), which visualizes the relationship between mean DSC score and FLOPs with bubble size encoding parameter count, shows ${\text{ZR}}^{2}$ ViM consistently occupying the upper-left quadrant. This position demonstrates superior segmentation accuracy at a comparable or lower computational cost. This performance contrasts sharply with that of other major architectural classes. For instance, CNN-based models like U-Net (Ronneberger et al., 2015) present an unfavorable trade-off, with our model reducing FLOPs by 72.8% for only a 12% increase in parameters. Transformer-based architectures such as TransUNet (Chen et al., 2024) and Swin-UNet (Cao et al., 2022) achieve high accuracy but at the cost of substantial computational and parameter overhead. Conversely, while other lightweight state space models like CC-ViM (Zhu et al., 2025) minimize complexity, they do not reach the same accuracy ceiling as ${\text{ZR}}^{2}$ ViM, which delivers state-of-the-art accuracy for a modest increase in computational cost. In addition to FLOPs and parameter count, we report a representative inference latency to provide practical insight into runtime efficiency. Inference time is measured on a single NVIDIA RTX 3080 GPU with batch size 1 and an input resolution of 256×256 , excluding data loading and preprocessing overhead. All models are warmed up prior to measurement, and the reported latency is averaged over multiple forward passes. As shown in Table 5, ${\text{ZR}}^{2}$ ViM maintains competitive inference latency despite incorporating cross-directional zigzag scanning and recursive connections, indicating that the proposed design does not introduce prohibitive runtime overhead in practice.

**TABLE 5:** Model complexity and inference latency comparison.

Type	Method	Params ↓ (M)	FLOPs ↓ (G)	Inference ↓ (ms)
CNN	UNet (Ronneberger et al., 2015)	34.50	65.52	28.87
	UNet++ (Zhou et al., 2018)	**9.26**	34.65	18.79
	Att-UNet (Oktay et al., 2018)	34.87	66.63	29.49
Transformer	TransUNet (Chen et al., 2024)	109.54	56.66	48.65
	TransFuse (Zhang et al., 2021)	43.40	47.28	28.83
	TC-Net (Dong et al., 2022)	33.71	33.56	29.02
	Swin-Unet (Cao et al., 2022)	82.30	67.30	34.82
	MISSFormer (Huang et al., 2022)	42.46	27.36	23.22
SSM	VM-UNet (Ruan et al., 2024)	26.35	21.38	26.73
	CC-ViM (Zhu et al., 2025)	23.56	14.45	**18.57**
	Swin-UMamba (Liu J. et al., 2024)	60.18	68.00	30.71
	SliceMamba (Fan et al., 2025)	20.53	16.52	25.45
	SA-UMamba (Liu et al., 2025)	43.45	24.72	21.78
	AEMMamba (Dong et al., 2025)	52.96	39.31	29.66
RWKV	Zig-RiR (Chen et al., 2025)	24.58	**12.45**	24.27
	HER-Seg (Xu et al., 2025)	25.21	14.48	22.71
	ZR^2^ViM (Ours)	38.66	17.84	22.35

Top results are highlighted in bold. FLOPs and Params are profiled with a unified input (1×3×256×256) . Inference latency is measured under the fixed setting described in the main text (RTX 3080, batch size 1, 256×256 , warm-up, averaged over multiple forward passes).

This advantageous balance between efficiency and effectiveness originates from a targeted architectural restructuring of the ViM framework. First, we replaced the standard S6 state space kernel with our SSR nested recursive state units. This design, coupled with NRC-driven local–global modeling under a unified scanning framework, minimizes the redundancy inherent in unstructured multi-branch approaches. Concurrently, the integration of the CZ-WKV module, featuring scalable m -step recursion, explicitly restores 2D spatial adjacency prior to sequence unrolling, thereby enhancing directional robustness. Notably, all components of ${\text{ZR}}^{2}$ ViM, including the cross-directional zigzag scanning and recursive operations, are implemented using standard PyTorch operators without relying on custom CUDA kernels or hardware-specific optimizations, facilitating consistent evaluation and fair comparison with Transformer-based and other baseline models.

### 4.6 Ablation study

To quantify the contribution of each key component in ${\text{ZR}}^{2}$ ViM, we conducted a series of module-wise ablation studies on the ISIC 2018 and Synapse multi-organ CT datasets. These two datasets were selected as representative testbeds because they cover two complementary segmentation regimes relevant to boundary-preserving modeling: ISIC 2018 is a 2D dermatoscopic lesion dataset with highly irregular contours, whereas Synapse is a multi-organ CT benchmark where boundary quality can be directly assessed using the boundary-sensitive HD95 metric in addition to DSC. Across all experiments, the network architecture and training hyperparameters were held constant, with only the specific module under investigation being modified. For skin-lesion segmentation (ISIC 2018), we report mIoU and DSC to measure overall accuracy. For multi-organ segmentation (Synapse), we report DSC and HD95 to assess boundary precision. This dual-metric design allows us to validate the proposed mechanisms under both region-overlap and boundary-focused criteria, while keeping the evaluation tailored to the distinct objectives of each task.

To clarify the performance–complexity trade-off of individual components, we additionally report the parameter count for each ablation variant in Tables 6–10. Notably, several ablation settings have identical or near-identical parameter counts, as these variants keep the overall network configuration fixed and only modify the target mechanism (e.g., scan scheduling, recursion depth, or path coordination) without introducing additional learnable layers. Therefore, the performance differences mainly reflect modeling capability rather than increased model capacity. Moreover, the full ${\text{ZR}}^{2}$ ViM model is evaluated on five public datasets across four imaging domains in the main experiments, and the ablations on these two representative datasets are used to explain why the overall gains occur.

**TABLE 6:** Contribution of key architectural components.

Methods	Params ↓ (M)	ISIC18		Synapse	
		DSC ↑	mIoU ↑	DSC ↑	HD95 ↓
ViM (Baseline)	26.35	89.90	82.08	78.73	23.09
ViM + SSR (Ours)	38.66	**92.22**	**85.65**	**83.04**	**15.68**

Top results are highlighted in bold. Params denote the total number of parameters (M). This table directly compares ViM (Baseline) and ViM + SSR, under the same settings to illustrate the accuracy–complexity trade-off.

**TABLE 7:** Efficacy of different spatial-mixing mechanisms.

Methods	Params ↓ (M)	ISIC18		Synapse	
		DSC ↑	mIoU ↑	DSC ↑	HD95 ↓
Bi-WKV	38.19	91.14	84.82	81.42	16.30
Re-WKV	38.19	91.31	85.08	81.59	16.19
Zigzag-WKV	38.19	91.39	85.17	82.19	16.04
CZ-WKV (Ours)	38.66	**92.22**	**85.65**	**83.04**	**15.68**

Top results are highlighted in bold. Params denote the total number of parameters (M). The compared variants have near-identical parameter counts because only the spatial-mixing mechanism is altered within a fixed architecture.

**TABLE 8:** Effectiveness of different scanning schemes.

Methods	Params ↓ (M)	ISIC18		Synapse	
		DSC ↑	mIoU ↑	DSC ↑	HD95 ↓
Sweep	38.19	91.64	84.38	81.08	16.77
CZ-Scan-1 (Single-Dir)	38.66	91.96	84.82	81.71	16.34
CZ-Scan-1 (Alt-Dir)	38.66	92.11	85.21	82.26	16.07
CZ-Scan-4 (Single-Dir)	38.66	92.15	85.37	82.58	15.86
CZ-Scan-4 (Alt-Dir)	38.66	**92.22**	**85.65**	**83.04**	**15.68**

Top results are highlighted in bold. Params denote the total number of parameters (M). Scan scheduling changes the traversal order and does not introduce additional learnable parameters.

**TABLE 9:** Sensitivity to recursion depth.

Setting	Params ↓ (M)	ISIC18		Synapse	
		DSC ↑	mIoU ↑	DSC ↑	HD95 ↓
m = 1	38.66	91.21	83.99	82.32	16.17
m = 2	38.66	91.35	84.22	82.58	16.03
m = 3	38.66	91.58	84.60	82.82	15.84
**m = 4**	38.66	**92.22**	**85.65**	**83.04**	**15.68**
m = 5	38.66	91.80	84.96	82.72	15.81

Top results are highlighted in bold. Params denote the total number of parameters (M). Varying the recursion depth m changes the number of recursion steps but does not add learnable parameters.

**TABLE 10:** Impact of the NRC structure.

Methods	Params ↓ (M)	ISIC18		Synapse	
		DSC ↑	mIoU ↑	DSC ↑	HD95 ↓
w/o NRC-Inner	37.89	91.08	83.01	81.35	16.33
w/o NRC-Outer	37.89	91.44	83.53	82.03	16.05
NRC-Inner → DWConv	37.92	91.87	84.16	82.57	15.87
NRC (Ours)	38.66	**92.22**	**85.65**	**83.04**	**15.68**

Top results are highlighted in bold. Params denote the total number of parameters (M). Minor parameter differences arise from removing or substituting NRC, pathways (e.g., replacing the inner pathway with DWConv).

#### 4.6.1 Efficacy of the SSR core operator

To determine if the proposed SSR operator mitigates spatial information loss during the 2D-to-1D sequence conversion, we compared its performance against the native S6 operator from the original ViM framework. As shown in Table 6, replacing the standard S6 operator with SSR yields substantial performance gains, accompanied by a moderate increase in parameter count. On the ISIC 2018 dataset, DSC and mIoU improved from 89.90% and 82.08%–92.22% and 85.65%, respectively. On the Synapse multi-organ CT dataset, DSC increased from 78.73% to 83.04%, while HD95 decreased from 23.09 mm to 15.68 mm. These results indicate that the performance improvements introduced by SSR are highly cost-effective relative to the added model capacity. The nested recursion and direction-aware mechanisms effectively suppress the inherent “stripe bias” of unidirectional scanning, better preserving spatial adjacency during sequence serialization and enabling more precise delineation of complex anatomical structures, particularly those with long boundaries or weak contrast.

#### 4.6.2 Comparison of attention-like kernels

To assess the efficacy of different spatial-mixing mechanisms for dynamic context aggregation, we compared several variants of the selective scan (WKV) kernel within a fixed architectural configuration. As presented in Table 7, the compared variants exhibit nearly identical parameter counts, isolating the effect of the spatial-mixing strategy itself. Performance improves progressively from Bi-WKV to Re-WKV and Zigzag-WKV, culminating in the highest accuracy with the proposed CZ-WKV. On the Synapse multi-organ CT dataset, CZ-WKV improves DSC by +1.62, +1.45, and +0.85 points over Bi-WKV, Re-WKV, and Zigzag-WKV, respectively, while also reducing HD95. This monotonic performance improvement underscores the effectiveness of cyclic recursion combined with zigzag scanning in enhancing long-range context aggregation without increasing model complexity.

#### 4.6.3 Impact of scan scheduling strategies

To assess the contribution of the scanning strategy to spatial context modeling, we compared the conventional Sweep scanning algorithm with our proposed Zigzag scheme in both unidirectional (Single-Dir) and alternating-direction (Alt-Dir) configurations. As detailed in Table 8, all Zigzag-based variants share identical parameter counts, allowing a fair comparison focused solely on scanning behavior. A consistent trend emerged across both datasets. First, all Zigzag scan variants (CZ-Scan) consistently outperformed the Sweep baseline, confirming that Zigzag paths better preserve spatial adjacency during sequence serialization. Second, within the Zigzag schemes, the Alt-Dir configuration consistently surpassed the Single-Dir one. On Synapse, for example, CZ–Scan-4 (Alt-Dir) increased DSC from 82.58% to 83.04% and reduced HD95 from 15.86 mm to 15.68 mm relative to its single-direction counterpart. This performance gain can be attributed to a “cross-path gap-filling” effect, wherein alternating scan directions correct for perceptual blind spots inherent in any single-direction scan. This mechanism significantly improves the continuity of segmented boundaries, particularly for intricate anatomical structures. These results empirically validate that the Zigzag scanning strategy enhances directional robustness while maintaining spatial coherence. Crucially, these findings demonstrate that substantial improvements in boundary continuity and directional robustness can be achieved solely through strategic scan scheduling, without increasing model capacity.

#### 4.6.4 Sensitivity to recursion depth

To determine the optimal recursion depth for modeling long-range feature interactions, we conducted a sensitivity analysis on the number of recursion steps, m. As reported in Table 9, all tested settings share identical parameter counts. Performance improved monotonically as m increased from 1 to 4, peaking at m = 4 (e.g., DSC on ISIC 2018 rose from 91.21% to 92.22%). However, performance slightly declined at m = 5 across both datasets. This finding suggests that a moderate increase in recursion efficiently expands the model’s effective receptive field, enabling more comprehensive aggregation of global contextual information. Conversely, excessive depth (m = 5) appears to introduce optimization challenges or result in saturated gains. Accordingly, we set m = 4 as the default, empirically optimal setting, which balances receptive-field expansion with training stability while preserving linear time complexity.

#### 4.6.5 Role of the NRC coordination mechanism

To elucidate the synergistic interaction between the inner and outer pathways of the NRC, we conducted systematic path removal and substitution experiments. As shown in Table 10, the ablation variants introduce only minor differences in parameter count, allowing a fair assessment of performance changes attributable to architectural coordination rather than increased model capacity. Despite these minimal parameter variations, removing or simplifying either pathway leads to pronounced performance degradation, revealing a clear performance hierarchy. Specifically, the complete nested structure achieves optimal performance, replacing the inner path with a depthwise convolution (DWConv) degrades accuracy, and ablating either the outer or inner pathway causes a more substantial drop. This gradient demonstrates that both pathways are indispensable and that their hierarchical coordination outperforms simpler parallel or partially ablated designs. These results suggest a functional division of labor within the NRC. The inner pathway focuses on restoring fine-grained local details, while the outer pathway leverages these cues to enhance global structural coherence. The “inside-out” residual injection mechanism is therefore essential for effectively integrating local and global context, enabling the nested architecture to deliver improved performance without relying on increased model capacity.

### 4.7 Discussion

Conventional segmentation frameworks, including those based on CNNs, Transformers, and SSMs (Ronneberger et al., 2015; Han et al., 2023; Liu Y. et al., 2024), universally rely on serializing two-dimensional feature maps into one-dimensional sequences. However, this serialization process disrupts the inherent spatial adjacency critical for accurate boundary localization, particularly in medical images where anatomical structures are elongated, tortuous, or have low contrast. This disruption manifests as stripe-like artifacts and inconsistencies along fine boundaries, resulting in higher (worse) HD95 scores on datasets like Synapse and fragmented lesion contours in ISIC. Consequently, the serialization bottleneck, rather than model capacity, emerges as the primary factor limiting boundary fidelity in these architectures.

To address this limitation, ${\text{ZR}}^{2}$ ViM restores two-dimensional spatial adjacency by introducing SSR and the CZ-WKV. By integrating direction-aware recursive updates with a zigzag-based sequence modeling strategy, ${\text{ZR}}^{2}$ ViM mitigates the anisotropy induced by conventional scanning methods. This dual approach enhances long-range dependency learning while preserving spatial adjacency during feature unfolding, ensuring a consistent and stable boundary representation. This is particularly effective for structures with irregular or elongated morphologies, as demonstrated in our experiments.

#### 4.7.1 Clinical significance

The model’s enhanced boundary preservation and robustness for low-contrast or slender anatomical structures hold substantial clinical value. Accurate delineation of irregular skin lesions enables more reliable tumor burden estimation, while clearer breast mass boundaries can reduce diagnostic uncertainty in ultrasound imaging. Furthermore, more consistent multi-organ segmentation is crucial for improving the precision of radiotherapy planning and preoperative assessments. By reducing boundary fragmentation and improving regional continuity, ${\text{ZR}}^{2}$ ViM can potentially decrease the manual correction workload for radiologists and enhance the reliability of downstream quantitative analyses.

#### 4.7.2 Limitations and future directions

Despite its strong performance, ${\text{ZR}}^{2}$ ViM has several limitations that suggest avenues for future research. First, the model exhibits slightly reduced accuracy on small or highly variable organs (e.g., the pancreas and gallbladder), highlighting the need for more powerful fine-grained feature modeling. Second, although the m -step recursive updates in CZ-WKV enhance directional context aggregation, they yield diminishing returns with increasing recursion depth; adaptive or data-driven recursion strategies could better balance computational cost and performance. Third, while zigzag scanning effectively restores spatial adjacency, its multi-path scheduling increases architectural complexity. More lightweight or learnable scanning schemes could reduce this structural overhead while preserving directional robustness.

Moreover, our current design primarily targets 2D feature serialization and thus does not explicitly model inter-slice correlations in volumetric data. Given the near-linear computational complexity of ${\text{ZR}}^{2}$ ViM, a promising direction is to extend the framework to direct 3D volumetric segmentation or video-based analysis. The proposed zigzag scanning strategy could be naturally generalized to 3D traversals (e.g., volumetric space-filling scheduling) to preserve adjacency along all three axes. This can be further combined with depth-wise recursive state propagation to capture cross-slice anatomical continuity without incurring the prohibitive memory cost of 3D Transformers. Similarly, for medical video segmentation (e.g., in ultrasound or endoscopic videos), the recursion-enhanced mechanism in SSR can be extended along the time axis by propagating the global state across frames while retaining spatial zigzag unfolding within each frame, enabling robust spatiotemporal consistency under motion and deformation.

Finally, large-scale evaluation on multi-center datasets representing diverse clinical scenarios is necessary to fully validate the generalizability of ${\text{ZR}}^{2}$ ViM and to facilitate its translation into real-world medical workflows.

## 5 Conclusion

This paper proposes a recursion-enhanced visual state space model, ${\text{ZR}}^{2}$ ViM, that addresses the loss of two-dimensional spatial adjacency and directional continuity arising from the serialization of medical images into one-dimensional sequences. Through its innovative SSR and CZ-WKV modules, ${\text{ZR}}^{2}$ ViM restores these critical spatial relationships. This approach enables the seamless integration of fine-grained local details with global semantic context while maintaining near-linear computational complexity. Evaluated across four imaging domains (dermatoscopic, ultrasound, endoscopic, and multi-organ CT) on five public datasets, ${\text{ZR}}^{2}$ ViM consistently outperforms representative CNN, Transformer, and SSM baselines. For instance, compared to the strong CC-ViM baseline, ${\text{ZR}}^{2}$ ViM improves the DSC by 2.32% on the ISIC 2018 dataset and reduces the HD95 by 2.15 mm on the Synapse multi-organ CT dataset. These quantitative improvements underscore the model’s superior capacity for preserving fine boundaries, maintaining structural continuity, and robustly segmenting challenging anatomical regions, such as those that are elongated or exhibit low contrast. By preserving spatial adjacency during sequence modeling and ensuring coherent information propagation along complex anatomical structures, ${\text{ZR}}^{2}$ ViM achieves robust long-range dependency modeling and precise boundary representation. This combination of high accuracy and computational efficiency makes the framework particularly valuable for clinical workflows requiring reliable boundary delineation, especially under resource constraints. Future research will extend this approach to 3D volumetric segmentation and explore adaptive or learnable scanning strategies. We also plan to investigate the benefits of large-scale pre-training, multimodal data integration, and rigorous multi-center clinical validation to further enhance the model’s robustness and generalizability for real-world medical applications.

## Data Availability

Publicly available datasets were analyzed in this study. This data can be found here: The datasets analyzed for this study can be found in the following public repositories. All links provided were accessible at the time of manuscript submission. 1. ISIC 2018 Dataset Repository/Platform: International Skin Imaging Collaboration (ISIC) Archive Direct URL: https://challenge.isic-archive.com/data/#2018 Reference/Citation: Codella, N., Rotemberg, V., Tschandl, P., Celebi, M. E., Dusza, S., Gutman, D., and Halpern, A. (2019). Skin lesion analysis toward melanoma detection 2018: A challenge hosted by the international skin imaging collaboration (ISIC). arXiv preprint arXiv:1902.03368. 2. BUSI (Breast Ultrasound Images) Dataset Repository/Platform: Cairo University Scholar Direct URL: https://scholar.cu.edu.eg/?q&equals;afahmy/pages/dataset Reference/Citation: Al-Dhabyani, W., Gomaa, M., Khaled, H., & Fahmy, A. (2020). Dataset of breast ultrasound images. Data in Brief, 28, 104863. 3. CVC-ClinicDB Dataset Repository/Platform: EndoVis (MICCAI Endoscopic Vision) Grand Challenge Direct URL: https://polyp.grand-challenge.org/CVCClinicDB/ Reference/Citation: Bernal, J., Sánchez, F. J., Fernández-Esparrach, G., Gil, D., Rodríguez, C., & Vilariño, F. (2015). WM-DOVA maps for accurate polyp highlighting in colonoscopy: Validation vs. saliency maps from physicians. Computerized Medical Imaging and Graphics, 43, 99-111. 4. Synapse Multi-Organ CT Dataset ◦ Repository/Platform: Synapse Direct URL: https://www.synapse.org/#!Synapse:syn3193805/wiki/217789 Reference/Citation: Landman, B., Xu, Z., Iglesias, J. E., Styner, M., Langerak, T., & Klein, A. (2015). MICCAI multi-atlas labeling beyond the cranial vault–workshop and challenge. In Proc. of the MICCAI Multi-Atlas Labeling Beyond Cranial Vault Workshop. The data supporting the findings of this study are publicly available from the repositories mentioned above. The corresponding accession numbers, DOIs, or direct links are provided in the list.

## References

[B1] Al-Dhabyani W. Gomaa M. Khaled H. Fahmy A. (2020). Dataset of breast ultrasound images. Data Brief 28, 104863. 10.1016/j.dib.2019.104863 31867417 PMC6906728

[B2] Bao J. Tan Z. Sun Y. Xu X. Liu H. Cui W. (2025). Deep ensemble learning-driven fully automated multi-structure segmentation for precision craniomaxillofacial surgery. Front. Bioeng. Biotechnol. 13, 1580502. 10.3389/fbioe.2025.1580502 40406586 PMC12094958

[B3] Bernal J. Sánchez F. J. Fernández-Esparrach G. Gil D. Rodríguez C. Vilariño F. (2015). Wm-dova maps for accurate polyp highlighting in colonoscopy: validation vs. saliency maps from physicians. Comput. Medical Imaging Graphics 43, 99–111. 10.1016/j.compmedimag.2015.02.007 25863519

[B4] Berseth M. (2017). Isic 2017-skin lesion analysis towards melanoma detection. 10.48550/arXiv.1703.00523

[B5] Cao H. Wang Y. Chen J. Jiang D. Zhang X. Tian Q. (2022). “Swin-unet: unet-like pure transformer for medical image segmentation,” in European conference on computer vision (Springer), 205–218. 10.1007/978-3-031-25066-8_9

[B6] Chan H.-P. Samala R. K. Hadjiiski L. M. Zhou C. (2020). Deep learning in medical image analysis. Adv. Exp. Med. Biol. 1213. 3–21. 10.1007/978-3-030-33128-3_1 32030660 PMC7442218

[B7] Chen J. Mei J. Li X. Lu Y. Yu Q. Wei Q. (2024). Transunet: rethinking the u-net architecture design for medical image segmentation through the lens of transformers. Med. Image Anal. 97, 103280. 10.1016/j.media.2024.103280 39096845

[B8] Chen T. Zhou X. Tan Z. Wu Y. Wang Z. Ye Z. (2025). Zig-rir: Zigzag rwkv-in-rwkv for efficient medical image segmentation. IEEE Trans. Med. Imaging 44, 3245–3257. 10.1109/TMI.2025.3561797 40244838

[B9] Codella N. Rotemberg V. Tschandl P. Celebi M. E. Dusza S. Gutman D. (2019). Skin lesion analysis toward melanoma detection 2018: a challenge hosted by the international skin imaging collaboration (isic). 10.48550/arXiv.1902.03368

[B10] Dong Y. Wang L. Li Y. (2022). Tc-net: dual coding network of transformer and cnn for skin lesion segmentation. Plos One 17, e0277578. 10.1371/journal.pone.0277578 36409714 PMC9678318

[B11] Dong X. Zhou B. Yin C. Liao I. Y. Jin Z. Xu Z. (2025). AEmmamba: an efficient medical segmentation model with edge enhancement. IEEE J. Biomed. Health Inf. 1–14. 10.1109/JBHI.2025.3572088 40397628

[B12] Duan Y. Wang W. Chen Z. Zhu X. Lu L. Lu T. (2024). Vision-rwkv: efficient and scalable visual perception with rwkv-like architectures. 10.48550/arXiv.2403.02308

[B13] Fan C. Yu H. Huang Y. Wang L. Yang Z. Jia X. (2025). Slicemamba with neural architecture search for medical image segmentation. IEEE J. Biomed. Health Inf. 29, 7446–7458. 10.1109/JBHI.2025.3564381 40279217

[B14] Gu A. Dao T. (2024). “Mamba: linear-time sequence modeling with selective state spaces,” in First conference on language modeling. Available online at: https://arxiv.org/abs/2312.00752 (Accessed May 31, 2024).

[B15] Han K. Wang Y. Chen H. Chen X. Guo J. Liu Z. (2023). A survey on vision transformer. IEEE Transactions Pattern Analysis Machine Intelligence 45, 87–110. 10.1109/TPAMI.2022.3152247 35180075

[B16] Hatamizadeh A. Tang Y. Nath V. Yang D. Myronenko A. Landman B. (2022). “Unetr: transformers for 3d medical image segmentation,” in 2022 IEEE/CVF Winter Conference on Applications of Computer Vision (WACV), 1748–1758. 10.1109/WACV51458.2022.00181

[B17] He K. Zhang X. Ren S. Sun J. (2016). “Deep residual learning for image recognition,” in Proceedings of the IEEE conference on computer vision and pattern recognition, 770–778. 10.1109/CVPR.2016.90

[B18] Huang X. Deng Z. Li D. Yuan X. Fu Y. (2022). Missformer: an effective transformer for 2d medical image segmentation. IEEE Transactions Medical Imaging 42, 1484–1494. 10.1109/TMI.2022.3230943 37015444

[B19] Isensee F. Jaeger P. F. Kohl S. A. Petersen J. Maier-Hein K. H. (2021). nnu-net: a self-configuring method for deep learning-based biomedical image segmentation. Nat. Methods 18, 203–211. 10.1038/s41592-020-01008-z 33288961

[B20] Jha D. Smedsrud P. H. Riegler M. A. Halvorsen P. De Lange T. Johansen D. (2019). Kvasir-seg: a segmented polyp dataset in International conference on multimedia modeling (Springer), 451–462. 10.1007/978-3-030-37734-2_37

[B21] Landman B. Xu Z. Igelsias J. Styner M. Langerak T. Klein A. (2015). “Miccai multi-atlas labeling beyond the cranial vault–workshop and challenge,” in Proc. MICCAI multi-atlas labeling beyond cranial vault—workshop challenge (Munich, Germany), 12. 10.48550/arXiv.2103.10504

[B22] Litjens G. Kooi T. Bejnordi B. E. Setio A. A. A. Ciompi F. Ghafoorian M. (2017). A survey on deep learning in medical image analysis. Med. Image Analysis 42, 60–88. 10.1016/j.media.2017.07.005 28778026

[B23] Liu Z. Lin Y. Cao Y. Hu H. Wei Y. Zhang Z. (2021). “Swin transformer: hierarchical vision transformer using shifted windows,” in Proceedings of the IEEE/CVF international conference on computer vision, 10012–10022. 10.1109/ICCV48922.2021.00986

[B24] Liu J. Yang H. Zhou H.-Y. Xi Y. Yu L. Li C. (2024). “Swin-umamba: Mamba-based unet with imagenet-based pretraining,” in International conference on medical image computing and computer-assisted intervention (Springer), 615–625. 10.1007/978-3-031-72114-4_59

[B25] Liu Y. Tian Y. Zhao Y. Yu H. Xie L. Wang Y. (2024). Vmamba: visual state space model. Adv. Neural Information Processing Systems 37, 103031–103063. 10.5555/3737916.3741189

[B26] Liu L. Huang Z. Wang S. Wang J. Liu B. (2025). Sa-umamba: spatial attention convolutional neural networks for medical image segmentation. PLoS One 20, e0325899. 10.1371/journal.pone.0325899 40504872 PMC12161529

[B27] Oktay O. Schlemper J. Folgoc L. L. Lee M. Heinrich M. Misawa K. (2018). Attention u-net: learning where to look for the pancreas. Available online at: http://arxiv.org/abs/1804.03999 (Accessed May 20, 2018).

[B28] Perazzi F. Pont-Tuset J. McWilliams B. Van Gool L. Gross M. Sorkine-Hornung A. (2016). “A benchmark dataset and evaluation methodology for video object segmentation,” in Proceedings of the IEEE conference on computer vision and pattern recognition, 724–732.

[B29] Ronneberger O. Fischer P. Brox T. (2015). “U-net: convolutional networks for biomedical image segmentation,”Int. Conf. Med. Image Computing Computer-Assisted Intervention, 9351. Springer, 234–241. 10.1007/978-3-319-24574-4_28

[B30] Ruan J. Li J. Xiang S. (2024). Vm-unet: vision mamba unet for medical image segmentation. New York, NY, USA: Association for Computing Machinery. Available online at:. 10.48550/arXiv.2402.02491

[B31] Shamshad F. Khan S. Zamir S. W. Khan M. H. Hayat M. Khan F. S. (2023). Transformers in medical imaging: a survey. Med. Image Analysis 88, 102802. 10.1016/j.media.2023.102802 37315483

[B32] Wang S. Li C. Wang R. Liu Z. Wang M. Tan H. (2021). Annotation-efficient deep learning for automatic medical image segmentation. Nat. Communications 12, 5915. 10.1038/s41467-021-26216-9 34625565 PMC8501087

[B33] Wu R. Liu Y. Ning G. Liang P. Chang Q. (2024). Ultralight vm-unet: parallel vision mamba significantly reduces parameters for skin lesion segmentation. Patterns 6, 101298. 10.1016/j.patter.2025.101298 41328156 PMC12664954

[B34] Xiao X. Lian S. Luo Z. Li S. (2018). “Weighted res-unet for high-quality retina vessel segmentation,” in 2018 9th international conference on information technology in medicine and education (ITME) (IEEE), 327–331. 10.1109/ITME.2018.00080

[B35] Xu Q. Lou Z. Li C. Li Y. He X. Berhanu T. F. (2025). Her-seg: holistically efficient segmentation for high-resolution medical images. 10.48550/arXiv.2504.06205

[B36] Yang Z. Li J. Zhang H. Zhao D. Wei B. Xu Y. (2025). Restore-rwkv: efficient and effective medical image restoration with rwkv. IEEE J. Biomed. Health Inf. 30, 1–14. 10.1109/JBHI.2025.3588555 40663663

[B37] Zhang Y. Liu H. Hu Q. (2021). “Transfuse: fusing transformers and cnns for medical image segmentation,” in International conference on medical image computing and computer-assisted intervention (Springer), 14–24. 10.1007/978-3-030-87193-2_2

[B38] Zhou Z. Rahman Siddiquee M. M. Tajbakhsh N. Liang J. (2018). “Unet++: a nested u-net architecture for medical image segmentation,” in Deep Learn. Med. Image Anal. Multimodal Learn. Clin. Decis. Support (Springer), 3–11. 10.1007/978-3-030-00889-5_1 PMC7329239 32613207

[B39] Zhou L. Xiao Z. Ning Z. (2023). “Rwkv-based encoder-decoder model for code completion,” in 2023 3rd International Conference on Electronic Information Engineering and Computer (EIECT) (IEEE), 425–428. 10.1109/EIECT60552.2023.10442108

[B40] Zhou P. Xie X. Lin Z. Yan S. (2024). Towards understanding convergence and generalization of adamw. IEEE Transactions Pattern Analysis Machine Intelligence 46, 6486–6493. 10.1109/TPAMI.2024.3382294 38536692

[B41] Zhu Y. Zhang D. Lin Y. Feng Y. Tang J. (2025). Merging context clustering with visual state space models for medical image segmentation. IEEE Trans. Med. Imaging 44, 2131–2142. 10.1109/TMI.2025.3525673 40030866

